# Epigenetic maintenance of adult neural stem cell quiescence in the mouse hippocampus via Setd1a

**DOI:** 10.1038/s41467-024-50010-y

**Published:** 2024-07-06

**Authors:** Ting Zhao, Yan Hong, Bowen Yan, Suming Huang, Guo-li Ming, Hongjun Song

**Affiliations:** 1 Department of Neuroscience and Mahoney Institute for Neurosciences, Perelman School of Medicine, University of Pennsylvania, Philedaphia, PA 19104 USA; 2 Department of Pharmacology and Therapeutics, College of Medicine, University of Florida, Gainesville, FL 32610 USA; 3 Division of Pediatric Hematology/Oncology, Department of Pediatrics, Pennsylvania State University College of Medicine, Hershey, PA 17033 USA; 4 Department of Cell and Developmental Biology, Perelman School of Medicine, University of Pennsylvania, Philedaphia, PA 19104 USA; 5 Department of Psychiatry, Perelman School of Medicine, University of Pennsylvania, Philedaphia, PA 19104 USA; 6 Institute for Regenerative Medicine, University of Pennsylvania, Philedaphia, PA 19104 USA; 7 Department of Neurosurgery, Perelman School of Medicine, University of Pennsylvania, Philedaphia, PA 19104 USA; 8 The Epigenetics Institute, Perelman School for Medicine, University of Pennsylvania, Philadelphia, PA 19104 USA

**Keywords:** Epigenetics and plasticity, Neural stem cells

## Abstract

Quiescence, a hallmark of adult neural stem cells (NSCs), is required for maintaining the NSC pool to support life-long continuous neurogenesis in the adult dentate gyrus (DG). Whether long-lasting epigenetic modifications maintain NSC quiescence over the long term in the adult DG is not well-understood. Here we show that mice with haploinsufficiency of *Setd1a*, a schizophrenia risk gene encoding a histone H3K4 methyltransferase, develop an enlarged DG with more dentate granule cells after young adulthood. Deletion of *Setd1a* specifically in quiescent NSCs in the adult DG promotes their activation and neurogenesis, which is countered by inhibition of the histone demethylase LSD1. Mechanistically, RNA-sequencing and CUT & RUN analyses of cultured quiescent adult NSCs reveal *Setd1a* deletion-induced transcriptional changes and many Setd1a targets, among which down-regulation of *Bhlhe40* promotes quiescent NSC activation in the adult DG in vivo. Together, our study reveals a Setd1a-dependent epigenetic mechanism that sustains NSC quiescence in the adult DG.

## Abstract (web summary)

Quiescence is required to maintain the neural stem cell pool to support life-long continuous adult neurogenesis in the hippocampus. Here the authors reveal an epigenetic mechanism sustaining neural stem cell quiescence in the adult mouse hippocampus.

## Introduction

Adult neurogenesis occurs throughout life in the dentate gyrus (DG) of the hippocampus in almost all mammals examined and contributes to specific brain functions, whereas its dysregulation has been implicated in a number of brain disorders^1–9^. In the adult mouse DG, radial glia-like cells (RGLs) are bona fide multipotent adult neural stem cells (NSCs)^10,11^ and an overwhelming majority of them reside in a state of reversible cell cycle arrest, named quiescence^12^. Once activated, RGLs give rise to new dentate granule cells and astrocytes and are often depleted^10,13–15^. Consequently, there is an age-dependent decline in the size of the adult NSC pool accompanied by reduced adult hippocampal neurogenesis^16–19^. Similarly, a number of genetic manipulations that promote precocious activation of quiescent RGLs in mice, such as deletion of FoxOs^20,21^ or Mfge8^22^, leads to premature depletion of the NSC pool during early postnatal stages, resulting in decreased adult hippocampal neurogenesis later in life. Given that quiescence is required to maintain life-long continuous neurogenesis^12,23^, a better understanding of the molecular mechanisms regulating adult NSC quiescence is of significant importance.

A growing body of evidence indicates that the quiescent state of RGLs is not a dormant state, but is actively regulated in the adult brain^12,24–28^. Extracellular signals from the local niche^29–32^ converge in RGLs, and downstream intrinsic factors, including metabolites^33–36^, signaling pathways^37^, cell cycle regulators^38^, and transcription factors^39–42^, mediate the effects of niche signals to balance RGL quiescence versus activation^12,23^. For example, a number of neuronal activity-dependent niche factors have been shown to dynamically regulate RGL quiescence^32^, such as GABA^30^ and sFRP3^29,43^. On the other hand, as a hallmark of adult NSCs, quiescence can be maintained over months and years before RGL reactivation^44^, and even upon deletion of factors such as PTEN^10^, Mfge8^22^, and GABAR_A_^30^ that promote quiescent RGL activation, the majority of RGLs remain quiescent. Therefore, beyond mechanisms that dynamically regulate quiescent RGL activation upon demand, there could exist a fundamental mechanism to ensure sustained adult NSC quiescence over the long term. Epigenetic mechanisms, including long-lasting DNA and histone modifications, are ideal candidates^45^. While epigenetic mechanisms have been shown to affect the identity and differentiation of adult NSCs and their progeny^46–52^, whether and how epigenetic mechanisms may regulate adult NSC quiescence in the DG is not well-understood^53–55^.

*Setd1a* encodes a histone methyltransferase that deposits mono-/di-/trimethylation epigenetic marks on the lysine 4 residue in histone 3 tails (H3K4me1/2/3)^56^. In humans, rare heterozygous loss-of-function variants in the *Setd1a* gene increase the risk of schizophrenia (SCZ) about 35-fold^57–59^. Several *Setd1a*^+/−^ mouse models have been generated to investigate how this SCZ-associated chromatin regulator affects brain functions and behaviors^60–62^. Some common findings include reduced levels of H3K4me3, disrupted expression of synaptic genes, defective synaptic transmission, aberrant morphology in neurons^60–62^, and behavioral abnormalities, such as deficits in spatial working memory and recognition memory^60,61^. Many of these deficits can be rescued with pharmacological inhibition of LSD1, a histone demethylase that counters the effect of *Setd1a*^60,63^, implying an epigenetic mechanism of *Setd1a* in these biological processes. To date, all published mouse studies have been focused on the role of *Setd1a* in the prefrontal cortex and striatum^60–62^, whereas its role in the hippocampus and neurogenesis has not been investigated. Here, we show that adult mice with *Setd1a* haploinsufficiency develop an enlarged DG. We further investigate the specific role of *Setd1a* in regulating adult hippocampal neurogenesis and identify an epigenetic mechanism that sustains NSC quiescence in the adult mouse DG.

## Results

### Increased number of dentate granule cells in adult mice with *Setd1a* haploinsufficiency

**Fig. 1:**
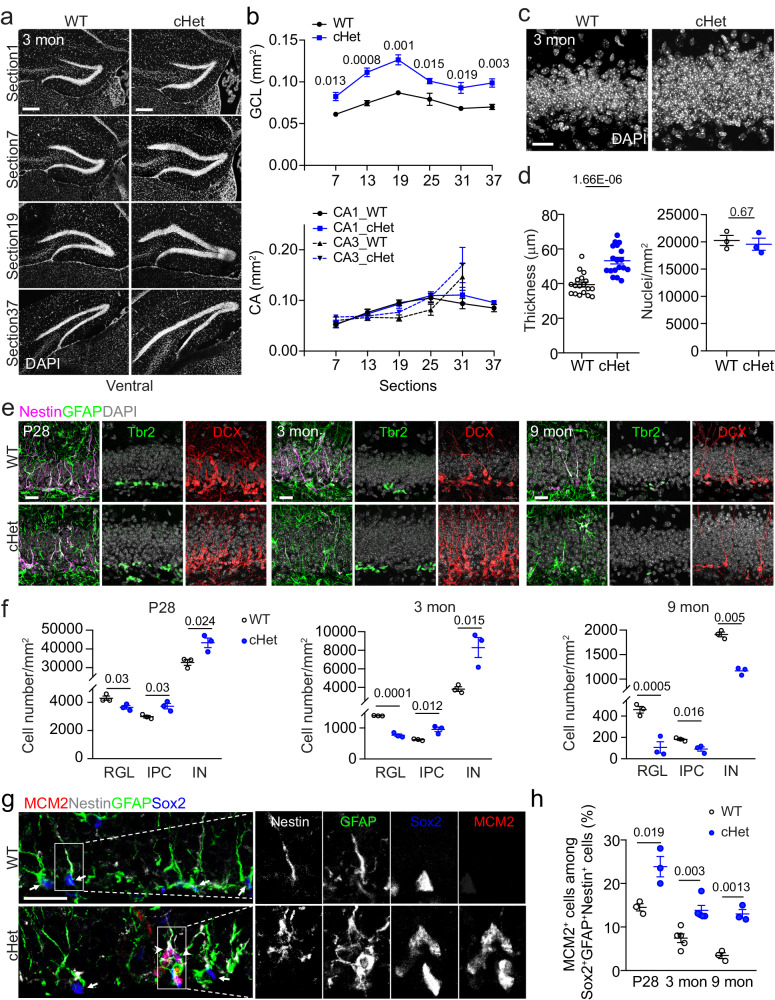
*Setd1a* haploinsufficiency results in an enlarged DG and elevated activation of DG quiescent RGLs in the adult mice. **a**, **b** Sample images (**a**; scale bars: 200 μm) and quantification of areas of the dentate granule cell layer (GCL), CA1 and CA3 neuronal layers of every 6th brain section along the dorsal-ventral axis of the hippocampus in 3-month-old WT and cHet mice (**b**). Values represent mean ± SEM (DG: *n* = 4/WT, 6/cHet; CA1: *n* = 4/WT, 3/cHet; CA3: *n* = 4/WT, 3/cHet). **c**, **d** Sample confocal images of DAPI staining of the suprapyramidal blade of the DG in 3-month-old WT and cHet mice (**c**; scale bar: 20 μm) and quantification of the thickness of GCL and densities of dentate granule cells (**d**). Each dot represents data from one section (left panel) or one mouse (right panel). Values represent mean ± SEM (*n* = 3/WT, 3/cHet). **e**, **f** Sample confocal images of immunostaining of various markers and DAPI (**e**; scale bars: 20 μm) and quantification of the densities of Nestin^+^GFAP^+^ radial glial-like neural stem cells (RGLs), Tbr2^+^ intermediate progenitor cells (IPCs) and DCX^+^ immature neurons (INs) in the DG of WT and cHet mice at P28, 3 months and 9 months (**f**). In **f**, each dot represents data from one mouse. Values represent mean ± SEM (*n* = 3/WT, 3/cHet for each condition). **g**, **h** Sample confocal images of immunostaining of RGL markers Nestin, GFAP and Sox2 and proliferation marker MCM2 RGLs in the DG of 3-month-old WT and cHet mice (**g**; scale bars: 20 μm) and quantification of percentages of MCM2^+^Nestin^+^GFAP^+^Sox2^+^ active RGLs among Nestin^+^GFAP^+^Sox2^+^ total RGLs in the DG of P28, 3-month-old and 9-month-old WT and cHet mice (**h**). Arrowheads mark MCM2^+^Nestin^+^Sox2^+^GFAP^+^ active RGLs and arrows mark MCM2^-^Nestin^+^Sox2^+^GFAP^+^ quiescent RGLs (**g**). In **h**, each dot represents data from one mouse. Values represent mean ± SEM (*n* = 5/WT, 5/cHet for 3 months and *n* = 3/WT, 3/cHet for P28 and 9 months). Two-tailed Student’s t-test is performed for statistical analysis and *P* values are listed. Source data are provided as a Source Data file.

We first crossed *Setd1a*^*fl/fl*^ mice^64^ with *Nestin-Cre* mice^65^ to delete *Setd1a* in the embryonic mouse brain. The efficacy of *Setd1a* deletion was confirmed by Western blotting with E17.5 brains (Supplementary Fig. 1a, b). Homozygous conditional knockout mice died by postnatal day 2 (P2) and heterozygous conditional knockout mice (hereafter, referred to as cHet) survived into adulthood. Interestingly, the DG was substantially enlarged in 3-month-old cHet mice compared to wildtype (*Setd1a*^*fl/+*^, WT) littermates (Fig. 1a, b). We measured the size of neuronal layers in the hippocampus in every 6^th^ section across the entire dorsal-ventral axis and found specific enlargement of the dentate granule cell layer, but not CA1 or CA3 neuronal layers in cHet compared to WT mice (Fig. 1a, b). Specifically, cHet mice exhibited increased layer thickness, but not density of dentate granule cells, compared to WT littermates (Fig. 1c, d). Further time course analysis showed no significant difference between WT and cHet mice in the size of the dentate granule cell layer at P14 and P28, but an increased size in cHet mice at 3 and 9 months (Fig. 1b and Supplementary Fig. 1c–h), indicating an adult-onset phenotype.

We next examined the cellular mechanism underlying the increased number of dentate granule cells in the adult cHet mice. The adult DG harbors a population of quiescent RGLs, which can be activated to give rise to new dentate granule cells throughout life^1,8,66^, and thus potentially contribute to the increased number of dentate granule cells. To examine adult hippocampal neurogenesis, we quantified densities of Nestin^+^GFAP^+^ RGLs, Tbr2^+^ intermediate progenitor cells (IPCs), and DCX^+^ immature neurons (INs) in the DG at P28, 3 months and 9 months. Compared to WT littermates, densities of IPCs and INs increased at P28 and 3 months, but decreased at 9 months in cHet mice, whereas the density of RGLs in cHet mice was lower at all time points examined with increasing differences over time (Fig. 1e, f). Next, we directly examined quiescent RGL activation and found an increased percentage of MCM2^+^Nestin^+^GFAP^+^Sox2^+^ active RGLs among Nestin^+^GFAP^+^Sox2^+^ total RGLs in the DG of cHet mice at P28, 3 months and 9 months compared to WT littermates (Fig. 1g, h).

Taken together, these results show that heterozygous *Setd1a* deletion in the embryonic nervous system results in the enlargement of DG in adulthood. Mechanistically, *Setd1a* haploinsufficiency leads to elevated activation of quiescent RGLs in the adult DG, resulting in an increased number of dentate granule cells and accelerated depletion of RGLs over time. Given the strong phenotype in heterozygous deletion animals (Fig. 1f–h), these findings suggest an indispensable role of *Setd1a* for the long term maintenance of RGL quiescence and the NSC pool in the adult DG.

### Elevated activation of quiescent RGLs in the postnatal DG upon induced *Setd1a* deletion

**Fig. 2:**
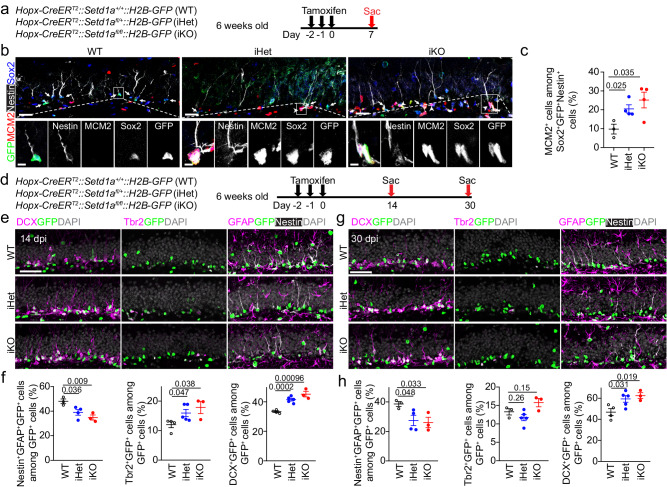
RGL-specific deletion of *Setd1a* promotes quiescent RGL activation and neurogenesis in the adult mouse DG. **a**–**c** Deletion of *Setd1a* specifically in RGLs promotes their activation in the adult DG. Shown in (**a**) is a schematic diagram of the experimental design. Shown in (**b**) are sample confocal images of immunostaining for various markers. Scale bars, 20 μm (inset, 5 μm). Arrowheads mark MCM2^+^Nestin^+^Sox2^+^GFP^+^ active RGLs and arrows mark MCM2^-^Nestin^+^Sox2^+^GFP^+^ quiescent RGLs. Shown in (**c**) is the quantification of the percentage of MCM2^+^Nestin^+^Sox2^+^GFP^+^ active RGLs among Nestin^+^Sox2^+^GFP^+^ total RGLs in the DG. Each dot represents data from one mouse. Values represent mean ± SEM (*n* = 3/WT, 4/iHet, 4/iKO; *P < 0.05). **d**–**h** Deletion of *Setd1a* in RGLs led to increased neurogenesis in the adult DG. Shown in (**d**) is a schematic diagram of the experimental design. Also shown are sample confocal images of immunostaining for various markers and DAPI at 14 (**e**) and 30 (**g**) days after the last tamoxifen injection (scale bars, 50 μm) and quantifications of percentages of Nestin^+^GFAP^+^GFP^+^ RGLs, Tbr2^+^GFP^+^ IPCs and DCX^+^GFP^+^ INs among total GFP^+^ cells in the DG of adult WT, iHet and iKO mice at 14 (**f**) and 30 (**h**) days after the last tamoxifen injection. In (**f**–**h**), each dot represents data from one mouse. Values represent mean ± SEM (14 days: RGLs: *n* = 3/WT, 4/iHet, 3/iKO; IPCs: *n* = 4/WT, 5/iHet, 3/iKO; INs: *n* = 4/WT, 5/iHet, 3/iKO; 30 days: RGLs: *n* = 3/WT, 4/iHet, 3/iKO; IPCs: *n* = 3/WT, 5/iHet, 3/iKO; INs: *n* = 4/WT, 5/iHet, 3/iKO). Two-tailed Student’s t-test is performed for statistical analysis and *P* values are listed. Source data are provided as a Source Data file.

In our *Nestin-cre*-based mice, *Setd1a* was deleted in all cells in the embryonic nervous system and there could be both non-cell autonomous effects and indirect effects of epigenetic inheritance from development on RGLs in the adult DG. To further support our model and examine the cell autonomous direct role of *Setd1a* specifically in quiescent RGLs, we generated sets of *Setd1a* inducible heterozygous and homozygous conditional deletion mice with matched controls, including *Hopx-CreER*^*T2*^::*Setd1a*^*fl/+*^*::H2B-GFP* (iHet), *Hopx-CreER*^*T2*^*::Setd1a*^*fl/fl*^*::H2B-GFP* (iKO), and *Hopx-CreER*^*T2*^*::Setd1*^*+/+*^*::H2B-GFP* (WT), by crossing mice with the *Setd1a*^*fl/fl*^ conditional allele with the *Hopx-CreER*^*T2*^ driver that specifically targets quiescent RGLs in the adult DG^44^ and has an inducible nuclear localized *H2B-GFP* reporter^67^. We injected 6-week-old mice with tamoxifen once daily for 3 days and analyzed the efficacy of *Setd1a* deletion in RGLs at 3 and 6 days after the last injection (dpi) by immunohistology (Supplementary Fig. 2a). Quantification of Setd1a expression showed reduced levels in RGLs of iHet mice at 3 and 6 dpi with further decreased levels in iKO mice, especially at 6 dpi (Supplementary Fig. 2b–e). We next examined RGL activation in these animals at 7 dpi (Fig. 2a). Quantification revealed a significant increase in the percentage of MCM2^+^Sox2^+^Nestin^+^GFP^+^ active RGLs among Sox2^+^Nestin^+^GFP^+^ total RGLs in the DG of both iHet and iKO mice compared to WT littermates (Fig. 2b, c). We then examined the consequence of elevated quiescent RGL activation at 14 and 30 dpi (Fig. 2d). At 14 dpi, quantification showed significantly increased percentages of Tbr2^+^ IPCs and DCX^+^ INs and decreased percentages of Nestin^+^GFAP^+^GFP^+^ RGLs among all GFP^+^ cells in the iHet and iKO mice compared to WT mice (Fig. 2e, f). At 30 dpi, there were still increased percentages of INs and decreased percentages of RGLs, but no difference in percentages of IPCs, among all GFP^+^ cells in the iHet and iKO mice compared to WT mice (Fig. 2g, h).

We also examined whether the role of *Setd1a* is specific to quiescent RGLs by analyzing NSCs in the developing DG. Our previous study has shown that *Hopx-CreER*^*T2*^ targets the population of NSCs in the developing DG that gradually transitions into quiescent RGLs from P7 to P14^28,44^, therefore we used the same set of animals. We injected tamoxifen once at P1 and examined the proliferation of Sox2^+^Nestin^+^GFP^+^ NSCs at P3, P7 and P14 (Supplementary Fig. 3a). We did not detect any differences in the percentage of MCM2^+^Sox2^+^Nestin^+^GFP^+^ NSCs among Sox2^+^Nestin^+^GFP^+^ total NSCs in the DG of WT, iHet and iKO mice at P3 and P7, when almost all NSCs were proliferating (Supplementary Fig. 3b–e). In contrast, at P14 when about 70% of NSCs were quiescent in the WT DG, the percentages of MCM2^+^Sox2^+^Nestin^+^GFP^+^ active NSCs among Sox2^+^Nestin^+^GFP^+^ total NSCs were increased in iHet and iKO mice (Supplementary Fig. 3f, g).

Together, results from this cell type-specific inducible system, particularly the significant effect in iHet mice, suggest a critical and highly sensitive cell autonomous role of *Setd1a* in maintaining RGL quiescence in the DG.

### Suppression of *Setd1a*-deficient quiescent RGL activation in the adult DG by LSD1 inhibition

**Fig. 3:**
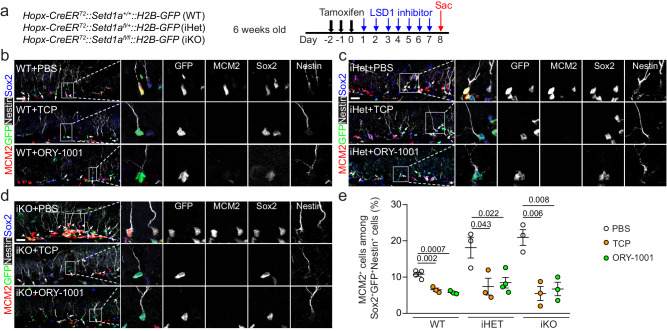
LSD1 inhibition counters *Setd1a* deficiency-induced RGL activation in the adult mouse DG. **a** A schematic diagram of the experimental design. **b**–**d** Sample confocal images of immunostaining for various markers. Scale bars: 20 μm. **e** Quantification of percentages of MCM2^+^Nestin^+^Sox2^+^GFP^+^ active RGLs among Nestin^+^Sox2^+^GFP^+^ total RGLs under the treatment of PBS (control) or LSD1 inhibitors (TCP or ORY-1001) in the DG of adult WT, iHet and iKO mice. Each dot represents data from one mouse. Values represent mean ± SEM (WT: *n* = 4/PBS, 3/TCP, 3/ORY-1001; iHet: *n* = 3/PBS, 3/TCP, 4/ORY-1001; iKO: *n* = 3/PBS, 3/TCP, 3/ORY-1001). Two-tailed Student’s t-test is performed for statistical analysis and *P* values are listed. Source data are provided as a Source Data file.

To investigate the molecular mechanism underlying *Setd1a*-dependent maintenance of RGL quiescence in the adult DG, we first asked whether Setd1a functions through an epigenetic mechanism, given that *Setd1a* is known to exert functions independent of its H3K4 methyltransferase activity, such as mediating DNA damage responses^68^. LSD1, a histone demethylase for H3K4me1/2, is known to counteract the H3K4 methyltransferase function of *Setd1a*^60,63^. For example, pharmacological LSD1 inhibitors TCP^69^ or ORY-1001^70,71^ rescued deficits in axon branching and abnormal behaviors of *Setd1a*^*+/-*^ mice^60^. TCP is a prototype LSD1 inhibitor that also inhibits two major isoforms of monoamine oxidase MAO-A and MAO-B^69^. ORY-1001 is a TCP derivative that exhibits most potent and selective LSD1 inhibition reported to date; ORY-1001 has an over 1000-fold selectivity for LSD1 compared to MAOs^60,70,71^. We injected tamoxifen once daily for 3 days into 6-week-old WT, iHet, and iKO mice followed by TCP or ORY-1001 injection once daily for 7 days, and then performed analysis one day after the last injection of LSD1 inhibitors (Fig. 3a). Immunohistological analysis confirmed that these two LSD inhibitors indeed elevated levels of H3K4me1/2 as well as H3K4me3 in RGLs in WT animals (Supplementary Fig. 4). Quantification showed that either TCP or ORY-1001 treatment decreased percentages of MCM2^+^Sox2^+^Nestin^+^GFP^+^ active RGLs among Sox2^+^Nestin^+^GFP^+^ total RGLs in both iHet and iKO mice (Fig. 3), supporting the notion that *Setd1a* maintains adult RGL quiescence via regulation of H3K4 methylation levels. Notably, LSD1 inhibition also decreased the level of quiescent adult RGL activation in WT mice (Fig. 3b, e). Collectively, these results suggest that *Setd1a* and LSD1, two epigenetic regulators, co-regulate the quiescent state of adult RGLs in the DG, but in the opposite manner.

### Epigenetically regulated transcriptomic changes in *Setd1a*-deficient adult NSCs

To further investigate the molecular mechanism underlying *Setd1a*-dependent maintenance of NSC quiescence, we turned to a culture model that mimics in vivo NSC quiescence by exposing adult NSC cultures^72^ to BMP4/FGF2^36,73,74^. We derived adult NSCs from the DG of 7 ~ 8-week-old *Setd1a*^*fl/fl*^*::Hopx-CreER*^*T2*^*::H2B-GFP* mice in the presence of EGF and FGF2 (Supplementary Fig. 5a). At 2 days after exposure to BMP4/FGF2 without EGF, almost all Sox2^+^ NSCs became Ki67^-^, indicative of quiescence (Supplementary Fig. 5a, b). Importantly, these quiescent adult NSCs were able to re-enter the cell cycle after replating in the presence of EGF/FGF2 without BMP4 (Supplementary Fig. 5a, b). We added lentivirus expressing Cre-GFP or GFP alone (control) to the quiescent adult NSC culture at 2 days after BMP4/FGF2 treatment (Supplementary Fig. 5c). About 80% of NSCs were infected by lentivirus as indicated by GFP expression and *Setd1a* levels were diminished at day 5 (3 days after viral infection) with Cre-GFP expression (Supplementary Fig. 5d–f).

**Fig. 4:**
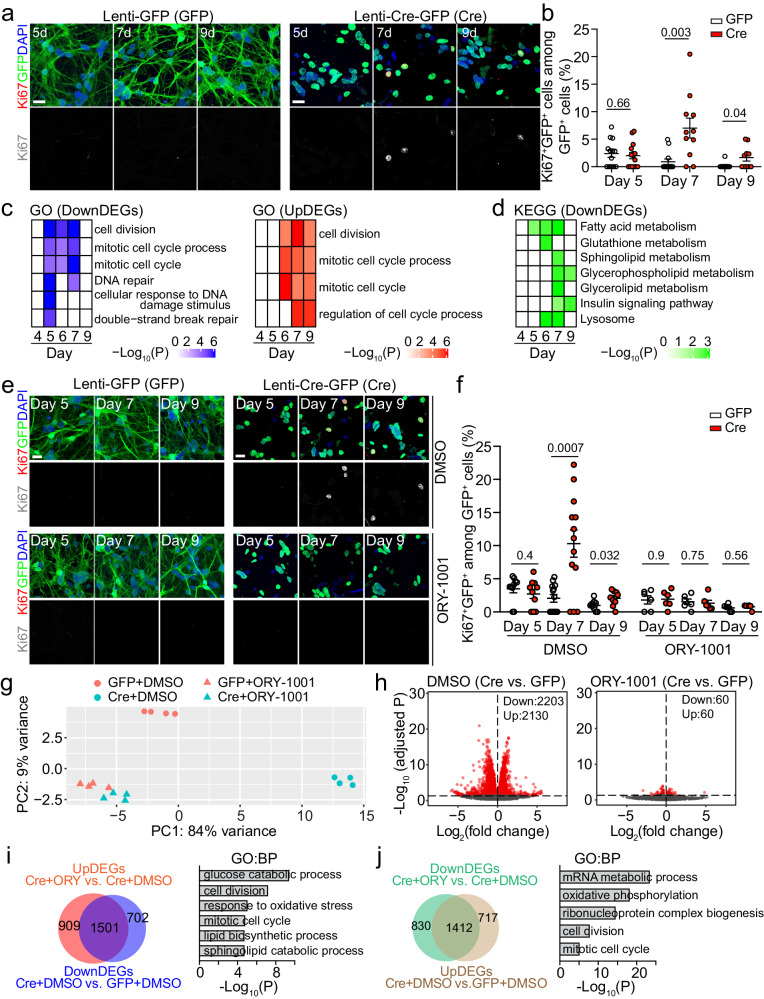
LSD1 inhibition rescues *Setd1a* deficiency-induced gene expression changes in cultured quiescent adult NSCs. **a**, **b** Sample confocal images of immunostaining of Ki67, GFP and DAPI (**a**; scale bars: 20 μm) and quantification of percentages of Ki67^+^GFP^+^ active NSCs among total GFP^+^ cells at days 5, 7 and 9 after lentivirus infection to express GFP or Cre-GFP in *Setd1a*-deficient NSCs at day 2. In **b**, each dot represents data from one image. Values represent mean ± SEM (*n* = 3 independent cultures for each condition). **c**, **d** Pathway analysis of differentially expressed genes upon *Setd1a* deletion in adult NSCs. Shown in (**c**) are heatmaps of GO analysis (biological process) of downregulated and upregulated genes in NSCs from days 4 to 9 with GFP or Cre-GFP expression, indicating dysregulated expression of cell cycle genes and DNA repair genes. Shown in (**d**) is a heatmap of KEGG pathway analysis of downregulated genes from days 4 to 9, revealing downregulated metabolic pathways. **e**, **f** Sample confocal images of immunostaining of Ki67, GFP, and DAPI (**e**; scale bars: 20 μm) and quantification of percentages of Ki67^+^GFP^+^ proliferating NSCs among total GFP^+^ cells under the treatment of LSD1 inhibitor ORY-1001 or DMSO as control at days 5, 7 and 9 with lentivirus to express GFP or Cre-GFP at day 2 (**f**). Values represent mean ± SEM (*n* = 3 independent cultures for each condition). **g**-**j** RNA-seq analysis of WT (GFP) and *Setd1a*-difficient (Cre-GFP) NSCs under the treatment of DMSO (control), or LSD1 inhibitor ORY-1001. Shown in (**g**) is a PCA plot of biological replicates. Shown in (**h**) are Volcano plots of differentially expressed genes under different conditions. Shown in (**i** and **j**) are Venn diagrams of genes that were dysregulated by *Setd1a* deletion and were rescued by LSD1 inhibitor treatment (left) and GO analysis (biological process) of these genes (right). Two-tailed Student’s t-test is performed for statistical analysis and *P* values are listed. Source data are provided as a Source Data file.

We next performed time course analyzes of these NSC cultures at days 4, 5, 6, 7 and 9 for immunostaining and RNA-seq (Supplementary Fig. 5c). We first examined whether our culture model could recapitulate the elevated activation of *Setd1a*-deficient quiescent RGLs in the adult brain in vivo. Indeed, deletion of *Setd1a* increased the percentage of Ki67^+^GFP^+^ proliferating NSCs among total GFP^+^ NSCs at days 7 and 9, but not at day 5 (Fig. 4a, b). We next examined differentially expressed genes (DEGs) between *Setd1a*-deficient (expressing Cre-GFP) and WT (expressing GFP) NSCs with RNA-seq from days 4 to 9 (Supplementary Dataset 1). GO analysis showed that the downregulated DEGs from *Setd1a*-deficient NSCs were related to cell cycle and DNA repair pathways between days 5 and 7, whereas cell cycle genes were upregulated in *Setd1a*-deficient NSCs between days 6 and 9 (Fig. 4c), consistent with the observation of elevated activation of *Setd1a*-deficient quiescent NSCs at days 7 and 9 (Fig. 4a, b). Our previous single-cell RNA-seq analysis revealed a shift in the energy source and metabolism when adult quiescent RGLs transit into active states in the DG in vivo^24^. Specifically, lysosomal and metabolic pathways enriched in quiescent adult RGLs, such as fatty acid, sphingolipid and glutathione metabolism, were downregulated upon adult RGL activation^24^. Similarly, KEGG pathway analysis of downregulated DEGs from *Setd1a*-deficient NSCs showed downregulated lysosomal and metabolism pathways, including fatty acid, glutathione and sphingolipid metabolism (Fig. 4d). These results suggest that *Setd1a* deficiency results in transcriptomic alterations in cultured quiescent adult NSCs that mimic quiescent adult RGL activation in the DG in vivo.

To assess whether Setd1a also functions through an epigenetic mechanism to maintain quiescence of cultured adult NSCs as in vivo (Fig. 3), we treated cultured *Setd1a*-deficient quiescent NSCs with the LSD1 inhibitor ORY-1001 from day 2 (Supplementary Fig. 5c). Indeed, ORY-1001 treatment suppressed the elevated activation of *Setd1a*-deficient quiescent NSCs in vitro (Fig. 4e, f). To obtain mechanistic insights into suppression effects of ORY-1001, we performed RNA-seq and found that ORY-1001 treatment dramatically reduced the number of DEGs between *Setd1a*-deficient and WT NSCs at day 7, from 4,333 DEGs between KO + DMSO and WT + DMSO conditions to only 120 DEGs between KO + ORY and WT + ORY conditions (Fig. 4g, h and Supplementary Dataset 1). As a result, most of the *Setd1a* deficiency-induced upregulated and downregulated DEGs were rescued by the ORY-1001 treatment (Fig. 4i, j). GO analysis revealed that these rescued DEGs are related to cell division, cell cycle and metabolism pathways (Fig. 4i, j).

Collectively, our in vitro results corroborated our in vivo findings and suggested that *Setd1a* deficiency-induced and epigenetically regulated transcriptomic changes in quiescent adult NSCs in vitro mimic in vivo adult RGL activation. This in vitro adult NSC model is therefore well suited for identification of *Setd1a* epigenetic targets that may mediate its effect on maintaining adult RGL quiescence in vivo.

### *Bhlhe40*, a *Setd1a* epigenetic target, maintains RGL quiescence in the adult DG

**Fig. 5:**
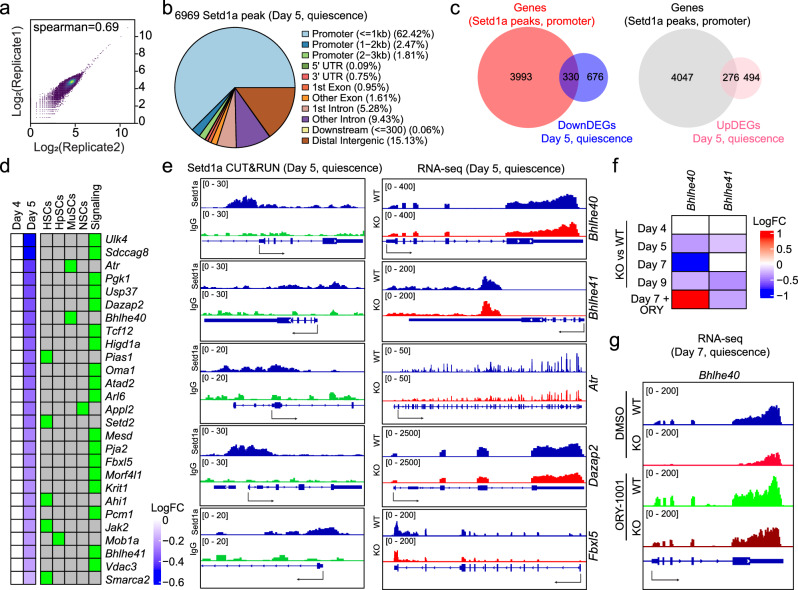
RNA-seq and CUT & RUN identifies *Setd1a* targets in quiescent adult NSCs. **a** Correlation of two independent *Setd1a* CUT & RUN analysis. **b** Distribution of CUT & RUN *Setd1a* peaks among genomic features in cultured quiescent NSCs at day 5. **c** Venn diagram showing the overlap between downregulated (left panel) and upregulated (right panel) genes in *Setd1a*-deficient compared to WT quiescent NSCs at day 5 from bulk RNA-seq data and genes with *Setd1a* peaks at the promoter regions in WT quiescent NSCs at day 5 from *Setd1a* CUT & RUN analysis. **d** Heatmaps showing *Setd1a*-target genes related to somatic stem cell quiescence. These representative genes were selected from 330 shared genes in the Venn diagram in (**c**), including genes reported to be important for maintaining quiescence of somatic stem cells in the adult mice and genes involved in the signaling pathways regulating quiescence of NSCs. HSCs, hematopoietic stem cells; HpSCs, hepatocyte stem cells; MuSCs, muscle stem cells; NSCs, neural stem cells. **e** Sequencing tracks of example *Setd1a* targets: *Bhlhe40, Bhlhe41, Atr, Dazap2* and *Fbxl5* from *Setd1a* CUT & RUN (left) and RNA-seq (right). **f**, **g** Heatmaps showing downregulation of *Bhlhe40* and *Bhlhe41* expression in *Setd1a*-deficient quiescent NSCs at indicated time points (**f**) and RNA-seq track of *Bhlhe40* loci under different conditions (**g**). Note that downregulation of *Bhlhe40* expression in *Setd1a-*deficient quiescent NSCs was rescued by ORY-1001 treatment at day 7.

To identify *Setd1a*-target genes, we performed a *Setd1a* CUT & RUN assay of cultured WT quiescent adult NSCs at day 5 and identified 6969 Setd1a binding sites (Fig. 5a, b and Supplementary Dataset 2). About 66% of the *Setd1a* peaks were localized at promoter regions, which is consistent with the known enrichment of H3K4me3 epigenetic marks at active promoters^75^ (Fig. 5b). To identify potential quiescence-related genes directly regulated by *Setd1a*, we compared genes harboring Setd1a peaks at promoters with DEGs in *Setd1a*-deficit NSCs from our RNA-seq data at day 5, a time point just before the elevated activation of *Setd1a*-deficient quiescent NSCs (Fig. 4a, b). We found 330 overlapped genes with downregulated DEGs and 276 overlapped genes with upregulated DEGs (Fig. 5c and Supplementary Dataset 2). Given that *Setd1a* is associated with active promoters and transcription activation^75^, we focused on 330 overlapped genes among downregulated DEGs and our further analysis identified 27 genes whose downregulation may potentially regulate somatic stem cell quiescence based on published studies (Fig. 5d). For example, deletion of either *Bhlhe40* or *Atr* has been reported to activate quiescent muscle stem cells in adult mice^76,77^ (Fig. 5d). A number of genes, such as *Dazap2*, *Fbxl5 and Pgk1*, have been reported to be involved in key signaling pathways regulating quiescence of somatic stem cells, such as Wnt, mTOR and PTEN pathways^78–80^ (Fig. 5d). In particular, our previous single-cell RNA-seq analysis revealed downregulation of *Bhlhe41* during RGL transition from quiescence to activation in the adult DG^24^. *Bhlhe40* is a paralogous gene of *Bhlhe41*, and *Bhlhe40*/*41* form a homodimer or a heterodimer to function as transcriptional repressors^81^. In cultured quiescent adult NSCs, the expression level of *Bhlhe40* was much higher than that of *Bhlhe41* (Supplementary Fig. 6a). While no studies so far have reported the role of *Bhlhe40*/*41* in the maintenance of adult NSC quiescence, depletion of *Bhlhe40* has been shown to activate quiescent muscle stem cells in adult mice^76^ and we found that downregulation of *Bhlhe40* in *Setd1a*-deficient NSCs was rescued by LSD1 inhibition (Fig. 5f, g). All together, these analyzes raised the possibility that *Bhlhe40* is one of the *Setd1a* targets that may regulate adult NSC quiescence.

**Fig. 6:**
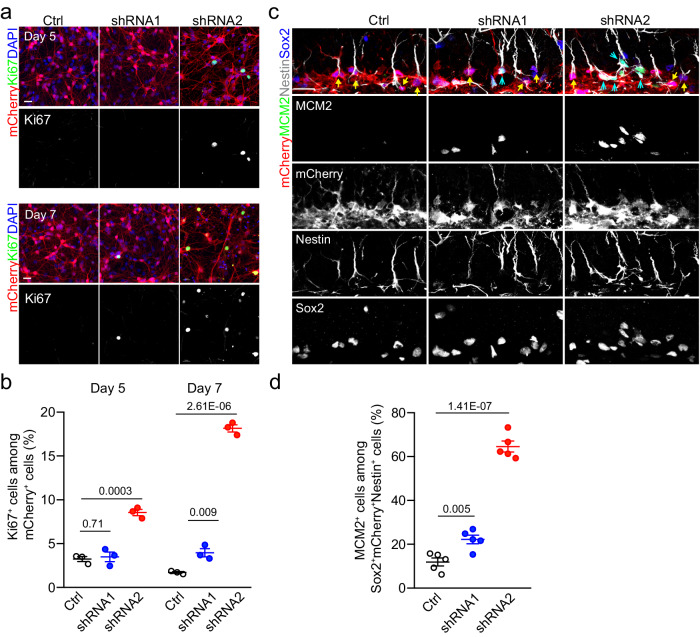
Knockdown of *Bhlhe40* promotes quiescent NSC activation in vitro and in the adult mouse DG. **a**, **b** Sample confocal images of immunostaining for mCherry, Ki67 and DAPI (**a**; scale bars: 20 μm) and quantification of percentages of Ki67^+^mCherry^+^ proliferating NSCs among total mCherry^+^ NSCs at days 5 and 7 in culture (**b**). In **b**, each dot represents data from one culture. Values represent mean ± SEM (*n* = 3 independent cultures for each condition). **c**, **d** Sample confocal images of immunostaining of various markers (**c**; scale bars: 20 μm) and quantification of percentages of mCherry^+^MCM2^+^Sox2^+^Nestin^+^ active RGLs among mCherry^+^Sox2^+^Nestin^+^ total infected RGLs in the DG of 2-month-old WT mice 7 days after lentiviral injection (**d**). Arrowheads mark infected MCM2^+^ active RGLs and arrows mark infected MCM2^-^ quiescent RGLs (**c**). In **d**, each dot represents data from one mouse. Values represent mean ± SEM (*n* = 5 mice for each condition). Two-tailed Student’s t-test is performed for statistical analysis and *P* values are listed. Source data are provided as a Source Data file.

To directly test this possibility, we expressed *Bhlhe40* shRNA with an mCherry reporter in cultured quiescent adult NSCs with lentivirus and qPCR analysis showed that *Bhlhe40* shRNA1 and shRNA2 decreased levels of *Bhlhe40* transcripts by approximately 30% and 70%, respectively (Supplementary Fig. 6b). Quantification further showed that *Bhlhe40* knockdown by either shRNA1 or shRNA2 led to increased percentages of Ki67^+^mCherry^+^ cells among all mCherry^+^ cells with shRNA2 exhibiting an earlier and larger effect than shRNA1, indicating that *Bhlhe40* maintains adult NSC quiescence in a dose-dependent manner (Fig. 6a, b). To corroborate these in vitro findings, we injected lentivirus into the DG of 2-month-old WT mice to knockdown *Bhlhe40* in adult RGLs. Quantification showed an increase in the percentage of MCM2^+^Sox2^+^Nestin^+^mCherry^+^ active RGLs among Sox2^+^Nestin^+^mCherry^+^ total infected RGLs for both shRNA1 and shRNA2 with a much stronger effect for shRNA2 (Fig. 6c, d).

Together, these results identified *Bhlhe40* as one major epigenetic target of *Setd1a* that maintains adult RGL quiescence in the DG.

## Discussion

Our comprehensive in vivo and in vitro studies with cellular, molecular and epigenetic analyzes identified a *Setd1a*-dependent epigenetic mechanism that sustains the quiescence of adult RGLs in the mouse DG. Beyond previous findings^12,23^ that provided insights into transcriptional and post-transcriptional control of adult NSC quiescence by genetic manipulations of niche factors^29–31^, cell cycle regulators^38^, transcription factors^39–42^, or metabolic enzymes^12^, our findings add an additional layer of epigenetic regulation to the quiescence control of adult NSCs and highlight the complexity of NSC quiescence regulation during life-long adult hippocampal neurogenesis. Importantly, the identified epigenetic mechanism potentially provides a foundation to ensure long term quiescence of adult NSCs, from which dynamic regulators can fine tune levels of adult NSC activation and neurogenesis upon demand. The significant effect of removing even one copy of *Seta1a* highlights the sensitive and indispensable role of such an epigenetic mechanism in sustaining NSC quiescence in the adult brain (Figs.  1f, h, 2c, and 3e,). Given that somatic stem cells in many adult tissue compartments are quiescent, our findings may have broad implications^82–84^.

Due to the long-lasting feature of epigenetic modifications, epigenetic mechanisms are ideal for sustained regulation, such as maintaining cell type identity throughout life^85,86^. Indeed, epigenetic mechanisms are known to regulate adult neurogenesis in the context of establishing cell type identity and regulating differentiation^46–48^. Here we identified an epigenetic mechanism via *Setd1a*-dependent H3K4 methylation for long term maintenance of NSC quiescence in the adult DG. A previous study showed that inhibition of histone deacetylases (HDACs) by valproic acid suppresses seizure-induced cell proliferation in the epileptic adult DG^54^. A further study showed that HDAC3 controls G2/M phase cell cycle progression of adult neural progenitors, therefore not quiescence maintenance^87^. Using a cell type-specific inducible system, we provided evidence for a cell autonomous and direct role of Sedt1a in maintaining adult NSC quiescence and further identified *Bhlhe40* as one of *Setd1a* targets that maintains adult NSC quiescence both in vitro and in vivo. Notably, the impact of *Bhlhe40*-downregulation on quiescent adult NSC activation appeared to be much stronger than *Setd1a* downregulation both in vitro and in vivo (Figs. 2c, 4b, 6b and 6d), indicating that *Bhlhe40* is a key factor maintaining adult RGL quiescence in the DG, whereas *Setd1a* is an upstream epigenetic regulator. Interestingly, Notch signaling is elevated in *Bhlhe40*-deficient muscle stem cells^76^. Notch signaling is essential for both proliferative and quiescent states of NSCs^88^, and promotes proliferation and quiescence through dynamic expression of different Notch effector proteins^89^. The downstream mechanism by which *Bhlhe40* regulates quiescence of adult RGLs remains to be determined. In the current study, we identified 330 *Setd1a* targets and a list of 27 potential targets that may regulate somatic stem cell quiescence (Fig. 5), but only validated the function of *Bhlhe40*. It is possible that *Setd1a* deficiency-induced activation of quiescent adult NSCs is a convergent effect of dysregulating multiple quiescence regulators. Our study provides a list of candidates for future studies to test their roles in regulating adult NSC quiescence in vitro and in vivo. A previous study has shown that depletion of *Bhlhe40* activates quiescent muscle stem cells in adult mice^76^. In addition, specific deletion of *Setd1a* in adult long term hematopoietic stem cells (LT-HSCs) results in G0-to-G1 transition and progression through cell cycle as well as increased numbers of Ki67^+^ LT-HSCs^90^. Whether the expression of *Bhlhe40* is downregulated in *Setd1a*-deficient LT-HSCs, which contributes to the activation of *Setd1a*-deficient LT-HSCs, remains to be examined. Together with these findings, our study points to a common mechanism that can maintain quiescence of adult somatic stem cells in different tissues.

*Setd1a* is one of six major H3K4 methyltransferases expressed in mammalian cells^91^. Whether other H3K4 methyltransferases, such as Setd1b and Mll1-4, also regulate quiescence of adult RGLs in the DG remains to be determined. However, activation of quiescent RGLs by heterozygous *Setd1a* deletion indicates that loss of *Setd1a* was not compensated by other H3K4 methyltransferases, thus the function of *Setd1a* on maintaining quiescence of adult RGLs is not redundant. Our results from pharmacological inhibition of LSD1 with TCP and ORY-1001 suggest that Setd1a-mediated maintenance of RGL quiescence in the adult DG is dependent on its H3K4 methyltransferase activity. A previous study reported that inhibition of LSD1 with polyamine analogs not only increased global levels of H3K4 methylation, but also H3K9ac, an epigenetic mark at active promoters^92,93^, at the promoters of *SFRP1*, *SFRP5*, *SFRP5* and *GATA5* in human colon carcinoma cells^94^. Although TCP specifically inhibits H3K4 demethylation without affecting the deacetylase activity^69^, we cannot rule out the possibility that TCP or ORY-1001 treatment increases H3K9ac levels at promoters of certain genes in quiescent RGLs.

*Setd1a* is an established risk gene for SCZ^57,58^. Different from previous studies that explored the effects of *Setd1a* deficiency on neuronal functions with mouse models or human induced pluripotent stem cell-derived neuronal models^60–62,95^, our study uncovered an unexpected role of *Setd1a* in the long-term maintenance of NSC quiescence during adult hippocampal neurogenesis and showed that *Setd1a* haploinsufficiency, which has been used to model SCZ^60–62^, leads to reduced adult hippocampal neurogenesis later in life in mice (Fig. 1f). SCZ patients exhibit learning and memory deficits and studies have suggested deficits of adult hippocampal neurogenesis in SCZ^96–98^. It is interesting to speculate that deficits in *Setd1a*-mediated adult NSC maintenance and decreased adult hippocampal neurogenesis may occur later in life in SCZ patients with Setd1a mutations and contribute to their cognitive deficits.

## Methods

### Animals

All experimental procedures with mice used in this study were performed in accordance with protocols approved by the Institutional Animal Care and Use Committee of University of Pennsylvania. All mice were kept in cages with bedding materials and housed in a 14-hour light/10-hour dark cycle with regular food and water changes, an ambient temperature of 21–23 °C and humidity levels between 40% and 60% (protocol numbers: 806245, 806246). *Setd1a*^*fl/fl*^ mice were previously generated in Dr. Suming Huang’s lab^64^. *Nestin-Cre*^+/Tg^ mice (Jackson Laboratory; 003771) were used to generate conditional knockout mice. These mice are referred to as *Setd1a*^*fl/+*^ (WT) and *Nestin-Cre::Setd1a*^*fl/+*^ *(cHet)*. To generate *Setd1a* cell type-specific inducible knockout animals, we crossed *Setd1a*^*fl/fl*^ mice with *Hopx-CreER*^*T2*^*::H2B-GFP* mice^44^. *Hopx-CreER*^*T2*^*::H2B-GFP* mice were generated by crossing *Hopx-CreER*^*T2*^ knock-in mice (Jackson Laboratory; 017606) that harbored a tamoxifen-inducible *CreER*^*T2*^ fusion gene with a Cre-reporter mouse line *Rosa26*^*flox-stop-flox-H2B-GFP*^ mice (from lab of Dr. Z. Josh Huang) harboring loxP sites on either side of a STOP sequence and upstream of a fusion H2B-GFP protein cassette^67^. These mice are referred to as *Hopx-CreER*^*T2*^*::Setd1a*^*+/+*^*:: H2B-GFP* (WT), *Hopx-CreER*^*T2*^*::Setd1a*^*fl/+*^*::H2B-GFP* (iHet), and *Hopx-CreER*^*T2*^*::Setd1a*^*fl/fl*^*::H2B-GFP* (iKO). Both male and female mice were used for all experiments, and no obvious sex phenotype was observed in any of the experiments and data were combined.

### Primary mouse adult neural stem cells

Adult NSCs were derived from the DG of 7 ~ 8-week-old *Hopx-CreER*^*T2*^*::Setd1a*^*fl/fl*^*::H2B-GFP* mice and cultured in proliferation medium containing Neurobasal medium (GIBCO), 20 ng/ml FGF2 (Peprotech), 20 ng/ml EGF (Peprotech), 2% B27 (v/v, GIBCO), 1% Glutamax (GIBCO), and 1% Penicillin/Streptomycin, on culture dishes precoated with poly-D-lysine (Sigma) and laminin (Sigma) as previously described^72^. To induce quiescence, cells were first plated into the proliferation medium at a density of 35,000–65,000 cells/cm^2^, and after 16 h fresh medium was added without EGF and with 20 ng/ml BMP4 (EMSCO/FISHER) and 20 ng/ml FGF2 (quiescent medium). For reactivation, after 3 days in the quiescence medium, cells were passaged with Accutase (Thermo Fisher) and plated into a proliferation medium at a density of 35,000–65,000 cells/cm^2^.

### Antibodies

Anti-Nestin (Aves labs, Cat#NES, 1:500), Anti-GFAP (Dako, Cat# Z0334, 1:500), Anti-GFAP (Sigma, Cat# MAB360, 1:500), Anti-TBR2 (Abcam, Cat#ab183991, 1:200), Anti-DCX (EMD Millipore, Cat#AB2253, 1:200), Anti-DCX (Santa Cruz, Cat# sc-8066, 1:200), Anti-Mcm2 (BD, Cat#610701, 1:150), Anti-Sox2 (Invitrogen, Cat#14-9811-82, 1:150), Anti-GFP (Rockland, Cat#600101215, 1:500), Anti-mCherry (Biorbyt, Cat#orb11618, 1:500), Anti-Ki67 (BD Biosciences, Cat#550609, 1:150), Anti-*Setd1a* (Abcam, Cat#ab70378, 1:150), Anti-*Setd1a* (Santa Cruz, Cat#sc-515590, 1:150), Anti-α-Tubulin (Cell Signaling, Cat#3873 S, 1:5000), Anti-H3K4me1 (Abcam, Cat#ab8895, 1:150), Anti-H3K4me2 (Abcam, Cat#ab7766, 1:150), Anti-H3K4me3 (Abcam, Cat#ab8580, 1:150)

### Western blot analysis

Brain tissues were lysed in ice-cold RIPA buffer (Thermofisher) containing protease inhibitor cocktail (Sigma) and phosphatase inhibitors (Cell Signaling). The lysates were sonicated and subjected to SDS-PAGE (BIO-RAD). The proteins on the gel were transferred to a nitrocellulose membrane (BIO-RAD), which was then blocked with 5% milk/TBST for 1 h at room temperature. The blot was incubated with primary antibodies in 5% milk/TBST overnight at 4 °C. After 3 washes in TBST, the blot was incubated with HRP-conjugated secondary antibodies in 5% milk/TBST for 1 h at room temperature. After 3 washes in TBST, ECL (Thermofisher) was used to detect immunoreactive bands on the blot using an Amersham Imager 600. Primary antibodies are listed in the Supplementary Dataset 3. Quantification of bands was performed using ImageJ software.

### Tissue processing and immunohistology

For immunostaining of brain sections, animals were transcardially perfused with ice-cold PBS, followed by fresh ice-cold 4% paraformaldehyde in PBS, and brains were post-fixed overnight in the same fixative at 4 °C as previously described^99^. Samples were cryoprotected in 30% sucrose in PBS overnight at 4 °C, embedded in tissue freezing medium and sectioned coronally (35 μm thickness) on a Leica CM3050S cryostat. Brain sections were washed with PBS, and then blocked and permeabilized with the blocking solution (2% donkey serum, 3% Bovine serum albumin, and 0.3% Triton X-100 in PBS) for 1 h at room temperature, followed by incubation with primary antibodies diluted in the blocking solution at 4 °C overnight. After washing with PBS, secondary antibodies diluted in blocking solution were applied to the sections for 1 h at room temperature. Nuclei were visualized by incubating for 10 min with DAPI (BD Biosciences) in PBS. Brain sections were mounted with Aqua-Mount Mounting Medium (EMSCO/FISHER) and analyzed. Brain sections immunostained for Nestin and MCM2 underwent antigen retrieval where sections were incubated in 1× Target Retrieval Solution (Agilent Dako) at 95 °C for 20 min in a steamer, then room temperature for 20 min before primary antibody incubation. If GFP immunostaining was performed in conjunction with Nestin or MCM, then GFP primary and secondary antibody steps were completed prior to antigen retrieval. For immunostaining of cultured cells, cells were fixed with fresh 4% paraformaldehyde in PBS for 10 min. The following procedure was the same as the immunostaining for brain sections. All the antibodies used are listed in the Supplementary Dataset 3.

### Confocal microscopy and image processing

Images were taken as z stacks using a Zeiss LSM 780 confocal microscope (Carl Zeiss). 20×, 40× or 63× objective was used for imaging. All confocal images were blindly acquired between experimental and control groups under the same laser power and gain. Images were analyzed using ImageJ software.

A confirmed RGL in Figs. 1e, 2e, g needed to satisfy the following criteria: (1) is located in the SGZ of the DG; (2) has a distinct Nestin^+^GFAP^+^ radial process; (3) has a DAPI^+^ nucleus that is largely surrounded by GFAP immunostaining signal in the same focal plane; and (for Fig. 2, g) (4) has a GFP^+^ nucleus. A confirmed RGL in Figs. 2b, 3b–d and Supplementary Fig. 4a–c needed to satisfy the following criteria: (1) is located in the SGZ of the DG; (2) has a distinct Nestin^+^ radial process; (3) has a Sox2 ^+^GFP^+^ nucleus in the same focal plane. A confirmed RGL in Fig. 1g needed to satisfy the following criteria, (1) located in the SGZ of the DG; (2) has a distinct Nestin^+^GFAP^+^ radial process; (3) has a Sox2 ^+^ nucleus in the same focal plane. A confirmed RGL in Fig. 6c needed to satisfy the following criteria: (1) is located in the SGZ of the DG; (2) has a distinct Nestin^+^ radial process; (3) has a Sox2 ^+^ nucleus in the same focal plane; (4) has an mCherry^+^ cell body. A confirmed RGL in Supplementary Fig. 2b,d needed to satisfy the following criteria: (1) is located in the SGZ of the DG; (2) has a distinct Nestin^+^ radial process; (3) has a DAPI^+^GFP^+^ nucleus in the same focal plane.

### Tamoxifen injection

A stock solution of 66.67 mg/mL tamoxifen (Thomas Scientific) was prepared in a 5:1 solution of corn oil:ethanol at 37 °C with occasional vortexing until dissolved^44^. Tamoxifen was injected intraperitoneally with one injection daily for 3 days for 6-week-old mice (Fig. 2a, d and Supplementary Fig. 2a) and once for P1 mice (Supplementary Fig. 3a).

### TCP preparation and injection

Tranylcypromine (TCP, Sigma) was dissolved in PBS at a stock concentration of 0.75 mg/ml by vortexing. Mice at 17–20 g body weight were injected 2.7 mg/kg/day; mice at 21–28 g body weight were injected 3.0 mg/kg/day as previously described^60^. 6-week-old animals were intraperitoneally injected for 7 consecutive days following tamoxifen injection (Fig. 3a).

### ORY-1001 preparation and injection

ORY-1001 (Cayman Chemical) was dissolved in PBS at a stock concentration of 1 mg/ml by vortexing. Mice at 17–20 g body weight were injected 9.0 μg/kg/day; mice at 21–28 g body weight were injected 10.0 μg/kg/day as previously descrived^60^. 6-week-old animals were intraperitoneally injected for 7 consecutive days following tamoxifen injection (Fig. 3a).

### RNA-seq library preparation, sequencing and analyzes

RNA-seq libraries were prepared based on the SMART-seq2 method as previously described with minor modifications^100,101^. Briefly, for RT, 3.2 μl RNA (100 ng/μl) was combined with 0.25 μl RNase inhibitor (NEB) and 1 μl CDS primer (10 mM, 5ʹ-AAGCAGTGG TATCAACGCAGAGTACT30VN-3ʹ) in an 8-well PCR tube strip and incubated at 70 °C for 2 min and immediately placed on ice. Then, 2 μl of 5× SMARTScribe RT buffer (TaKaRa), 0.5 μl of DTT (100 mM). 0.3 μl of MgCl_2_ (200 mM), 1 μl of dNTPs (10 mM), 1 μl of TSO primer (10 μM, 5ʹ-AAGCAGTGGTATCAACGCAGAGTACATrGrGrG-3ʹ) 0.25 μl of RNase inhibitor (NEB), and 0.5 μl SMARTScribe reverse transcriptase (TaKaRa) was added to the reaction. RT was performed under the following conditions: 42 °C for 90 min, 10 cycles of 50 °C for 2 min and 42 °C for 2 min, 70 °C for 15 min and 4 °C indefinitely. For cDNA amplification, 2 μl of the RT reaction was combined with 2.5 μl of 10× Advantage 2 buffer (Takara), 2.5 μl of 2.5 mM dNTPs (Takara), 0.25 μl of 10 μM IS PCR primer (5ʹ-AAGCAGTGGTATCAACGCAGAGT-3ʹ), 17.25 μl nuclease-free water, and 0.5 μl Advantage 2 DNA polymerase (Takara). Thermocycling conditions were as follows: 94 °C for 3 min, 9 cycle of 94 °C for 15 s, 65 °C for 30 s, and 68 °C for 6 min, 72 °C for 10 min, and 4 °C indefinitely. Amplified cDNA was then purified using 0.8× AMPure XP beads (Beckman Coulter), eluted in nuclease-free water and quantified following the instructions of Qubit dsDNA HS assay kit (Thermo Fisher). For tagmentation of cDNA, 50 pg cDNA in 1μl nuclease-free water, 2.5 μl 2×TD buffer (20 mM Tris/PH 8.0, 10 mM MgCl_2_, and 16% PEG 8000) and 0.5 μl adapter-loaded Tn5 transposase (Lucigen) were mixed and incubated at 55 °C for 15 min and the reaction was terminated by adding 1.25 μl 0.2% SDS and incubated at room temperature for 10 min. Fragments were amplified by adding 16.75 μl nuclease-free water, 1 μl of Nextera i7 primer (10 mM), 1 μl of Nextera i5 primer (10 mM), and 25 μl KAPA HiFi hotstart readymix (EMSCO/FISHER). Thermocycling conditions were as follows: 72 °C for 5 min, 95 °C for 1 min, 14 cycles of 95 °C for 30 s, 55 °C for 30 s, and 72 °C for 30 s, 72 °C for 1 min, and 4 °C indefinitely. PCR products were then purified twice with 0.8× AMPure XP beads and eluted in nuclease-free water. Samples were quantified by qPCR (KAPA) and pooled at equal molar amounts. The average fragment size of the final library fragment was quantified by bioanalyzer (Agilent), and the final concentration of the library was determined by qPCR (KAPA). About 2.7 pmol DNA was loaded on NextSeq high output kit (75 cycles, Illumina) and subjected to a NextSeq 550 sequencer (Illumina). Raw sequencing data from RNA-seq were demultiplexed with bcl2fastq2 v2.17.1.14 (Illumina), and adapters were trimmed using Trimmomatic v0.32^102^. Alignments were made using STAR v2.5.2a^103^ to GENCODE mouse reference genome GRCm38. Only uniquely mapped reads were quantified at the gene level and summarized to gene counts using STAR-quantMode (Gene Counts), with multimapping and chimeric alignments discarded. Further analyzes were performed in R (v3.6.0). Differentially expressed genes were identified using edgeR^104^. Identified upregulated and downregulated gene lists were used for Gene Ontology (GO) enrichment and KEGG pathway analyzes using Metascape Database^105^. Fold changes of target differentially expressed genes were visualized in heatmaps by “ComplexHeatmap” package^106^ in R.

### Administration of LSD1 inhibitor into primary adult NSC cultures

LSD1 inhibitor, ORY-1001 (Cayman Chemical), was dissolved at 30 μM in 100% DMSO. For inhibiting LSD1 enzymatic activity in cultured quiescent NSCs, ORY-1001 was diluted to 15 nM in fresh quiescence medium. Quiescence medium without ORY-1001 was first added to primary NSC cultures to induce quiescence. After 48 h, medium was replaced with fresh quiescence medium containing 15 nM ORY-1001 as well as lentivirus expressing either GFP or GFP-Cre. Quiescence medium with 15 nM ORY-1001 was replaced every 2 days until cultures were harvested for immunostaining or RNA-seq experiments.

### CUT & RUN assay and bioinformatic analyzes

The *Setd1a* CUT & RUN samples were prepared with CUT & RUN kit (EpiCypher). Briefly, cultured quiescent NSCs were collected in a 1.5 ml tube containing wash buffer. Cells were captured with ConA beads and incubated with *Setd1a* antibody (Abcam: ab70378) overnight at 4 °C in antibody incubation buffer. After unbound antibodies were washed away, pAG-MNase was added and incubated for 10 min at RT. After washing, CaCl_2_ was added and incubated for 2 h at 4 °C, and the reaction was stopped by adding stop buffer. The protein-DNA complexes were released by incubating at 37 °C for 10 min. The supernatant was transferred to a new Lo-bound tube. DNA was then purified using a DNA cleanup kit. Sequencing libraries were prepared using the NEBNext Ultra II DNA library preparation kit for Illumina (New England Biolabs) according to the manufacturer’s instructions. Briefly, end repair was conducted at 20 °C for 30 min, followed by dA-tailing at 65 °C for 30 min. After adapter ligation, the DNA fragments were purified by 0.9× volume of SPRIselect beads (EMSCO/FISHER) followed by 14 cycles PCR amplification with NEBNext Ultra II Q5 Master Mix (New England Biolabs). The PCR products were cleaned up with 0.9× volume of SPRIselect beads. The CUT & RUN libraries were quantified using qPCR (KAPA) and pooled at equal molar amounts, and quality control was performed with the Bioanalyzer High Sensitivity DNA Analysis (Agilent). The libraries were sequenced on the NextSeq high output kit (75 cycles, Illumina). For the analysis of the CUT & RUN data, all the reads were trimmed, and then uniquely mapped to the mm10 reference genome using Bowtie2^107^. Low mapping quality reads were filtered by SAMtools^108^. Peak calling was performed using MACS2 with default parameters^109^. The intersected peaks defined in both replicates were considered high confidence peaks. The BAM files were converted to Bigwig format using deepTools^110^. The normalized read densities of merged replicates were visualized using the Integrative Genomics Viewer (IGV). Peak annotation was performed with ChIPseeker^111^.

### Plasmid constructs

For knockdown experiments for mouse *Bhlhe40*, the short hairpin RNA sequence was cloned into the lentiviral vector expressing mCherry under the control of the hPGK promoter and the specific shRNA under the control of U6 promoter (Addgene plasmid #128073). All shRNA sequences are listed in the Supplementary Dataset 3. The efficacy of each *Bhlhe40* shRNA was confirmed in cultured quiescent NSCs.

### Lentivirus preparation and stereotaxic injection in the adult DG

When HEK293T cells cultured on the 15 cm dish reached 80% confluence, pLV-eGFP^112^ (Addgene) or pLV-EGFP-Cre^113^ vector (Addgene) was co-transfected with pCMV-VSV-G (Addgene) and HIV-1 packaging plasmid Δ8.9^114^ into HEK293T cells with LipoD293 in vitro transfection reagent (SignaGen). For lentivirus expressing specific shRNA against mouse *Bhlhe40*, mCherry and shRNA were co-expressed under the control of the hPGK and U6 promoters, respectively, using the pLK0.1 mCherry plasmid (Addgene). After co-transfection, the culture medium was collected once every 24 h for three times. The lentivirus in the collected medium was concentrated by ultracentrifugation at 106,800 g (25,000 rpm) for 1.5 h at 4 °C, with the supernatant discarded and pellet reconstituted with PBS. The titer of virus was measured by counting the number of infected cell clusters in a serial dilution experiment. Two-month-old mice were anesthetized and placed in a David Kopf Instruments stereotaxic frame (model 1900) equipped with a digital manipulator and a UMP3-1 Ultra pump. Mice were kept deeply anesthetized as assessed by monitoring pinch withdrawal and respiration rate. Viral injections were targeted to the dentate gyrus (A/P: −2.0 mm; M/L: 1.5 mm; D/V: −2.6 mm). The injections were performed at a rate of 0.2 μl/min. The needle was left in place for 10 min after each injection to minimize upward flow of viral solution after raising the needle.

### RNA extraction and qPCR

Cell cultures were dissolved in 1 ml Trizol (Thermo Fisher) for 15 min and 200 μl chloroform was then added. After centrifugation 12000 g for 15 min at 4 °C, about 400 μl supernatant was carefully taken out and mixed with the same volume of ethanol, and the mixture was transferred to Zymo-Spin^TM^ IC Column in a collection tube from the kit of Zymo RNA clean & concentrator kit (Zymo Research). After centrifugation, 400 μl RNA Prep buffer, 700 μl RNA wash buffer and 400 μl RNA wash buffer was sequentially added to the column with each procedure followed by centrifugation. Finally, about 15 μl nuclease-free water was added to elute the column to collect the RNA sample. RNA concentration and quality were assessed using a Nanodrop 2000. Subsequently, about 1 μg RNA was used for reverse transcription (RT) to synthesize cDNA with the SuperScript III First-Strand Synthesis System (Thermo Fisher). Quantitative RT-PCR was then performed using SYBR Green (Thermo Fisher) and the StepOnePlus Real-Time PCR system (Applied biosystems). Quantitative levels for all genes were normalized to the house-keeping gene *GAPDH* and expressed relative to the relevant control samples. All primer sequences are listed in the Supplementary Dataset 3.

### Quantification and Statistical analyzes

Data in figure panels reflect several independent experiments performed on different days. Unpaired two-tailed Student’s t-tests were performed with GraphPad Prism. Quantitative results are expressed as the means ± SEM. A *P* value < 0.05 was considered significant. Statistical significance level was set as follows: *if *P* < 0.05, **if *P* < 0.01, ***if *P* < 0.001, n.s: *P* > 0.05.

### Reporting summary

Further information on research design is available in the Nature Portfolio Reporting Summary linked to this article.

### Supplementary information

Supplementary information

Peer Review File

Description of Additional Supplementary Files

Supplementary Dataset 1

Supplementary Dataset 2

Supplementary Dataset 3

Reporting Summary

### Source data

Source Data

## Data Availability

The RNA-seq and CUT & RUN data generated in this study have been deposited in the NCBI’s Gene Expression Omnibus database under accession code GSE250278. Source data are provided with this paper. No custom software algorithms were generated. Any additional information required to reanalyze the data reported in this paper is available from the corresponding authors upon request. Source data are provided with this paper.
