# Supplementary material for: Epigenetic maintenance of adult neural stem cell quiescence in the mouse hippocampus via Setd1a
Source: Nat Commun. 2024 Jul 6;15:5674. doi: 10.1038/s41467-024-50010-y (PMC11227589; doi:10.1038/s41467-024-50010-y)

SUPPLEMENTARY FIGURES

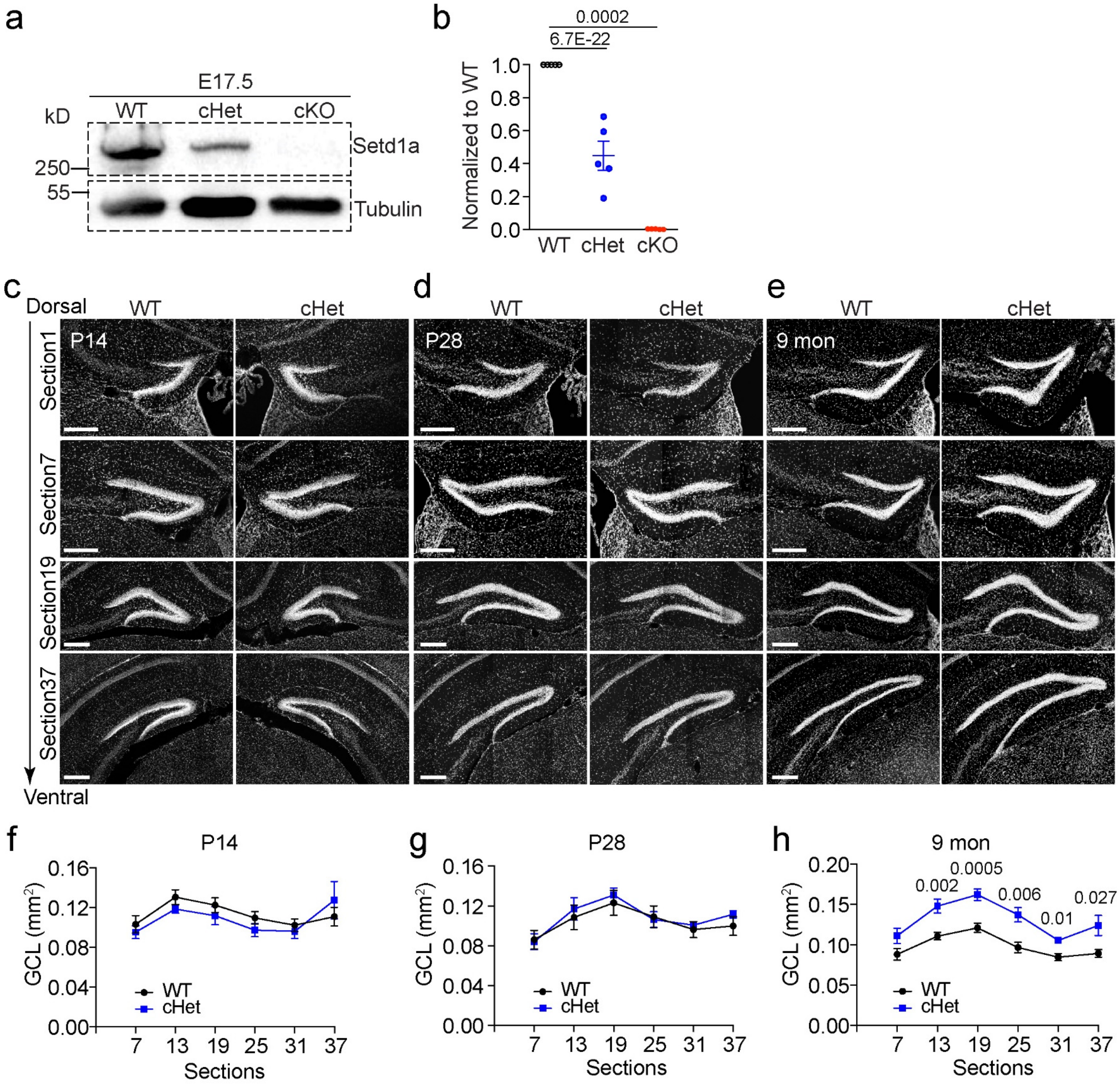

Supplementary Fig. 1. Time-dependent impact of *Setd1a* haploinsufficiency on the size of the dentate gyrus in mice. a, b Sample western blotting images of Setd1a in WT, cHet and cKO brains at E17.5 (**a**) and quantification of Setd1a levels (**b**). The ratio of Setd1a signal intensity over tubulin signal intensity was first calculated and then normalized to the value of WT. Each dot represents the result from one experiment. Values represent mean ± SEM (n = 5/WT, 5/cHet, 5/cKO). **c-h** Sample confocal images of DAPI staining (**c-e**; scale bars: 200 μm) and quantification of the areas of WT and cHet dentate granule cell layers every 6th brain section from dorsal to ventral hippocampus at P14, P28 and 9 months (**f-h**). Values represent mean ± SEM (P14: n = 4/WT, 4/cHet; P28: n = 4/WT, 3/cHet; 9 months: n = 8/WT, 8/cHet). Two-tailed Student's t test is used and the P values are shown. Source data are provided as a Source Data file.

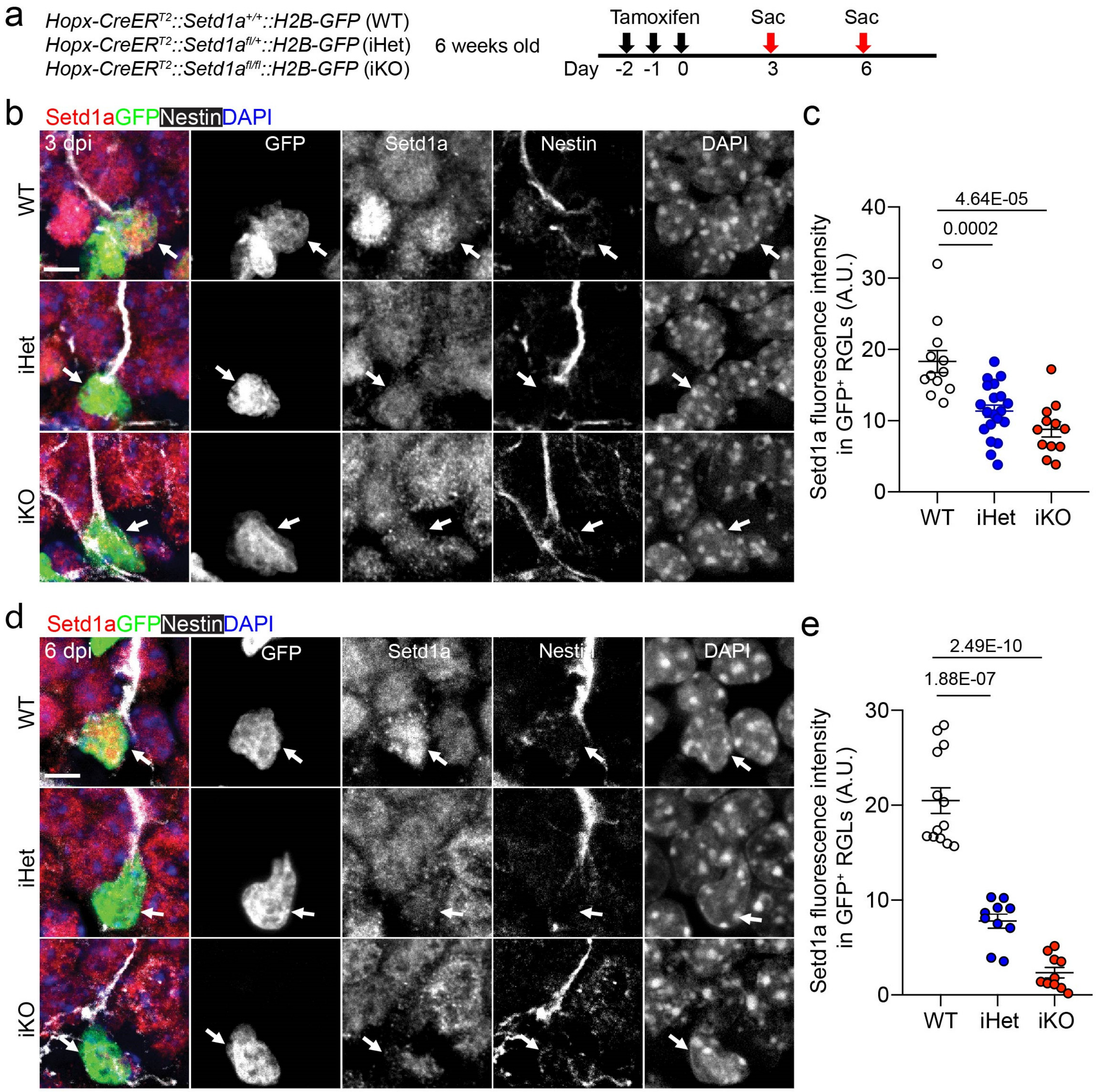

Supplementary Fig. 2. Confirmation of *Setd1a* deletion in RGLs in the adult DG. a A schematic diagram of the experimental design. **b-e** Sample confocal images of immunostaining for GFP, Setd1a, Nestin and DAPI at 3 (**b**) and 6 (**d**) days after the last tamoxifen injection (scale bars: 5 μm) and quantification of Setd1a protein levels in RGLs in the DG of WT, iHet and iKO mice at 3 (**c**) and 6 (**e**) days after the last tamoxifen injection. In (**c, e**), each dot represents data from one RGL. Values represent mean ± SEM (3 days: n = 12 RGLs/WT, 20 RGLs/iHet, 12 RGLs/iKO; 6 days: n = 13 RGLs/WT, 10 RGLs/iHet, 10 RGLs/iKO; from n = 3 animals each condition). A.U.: Arbitrary Unit. Two-tailed Student's t test is used and the P values are shown. Source data are provided as a Source Data file.

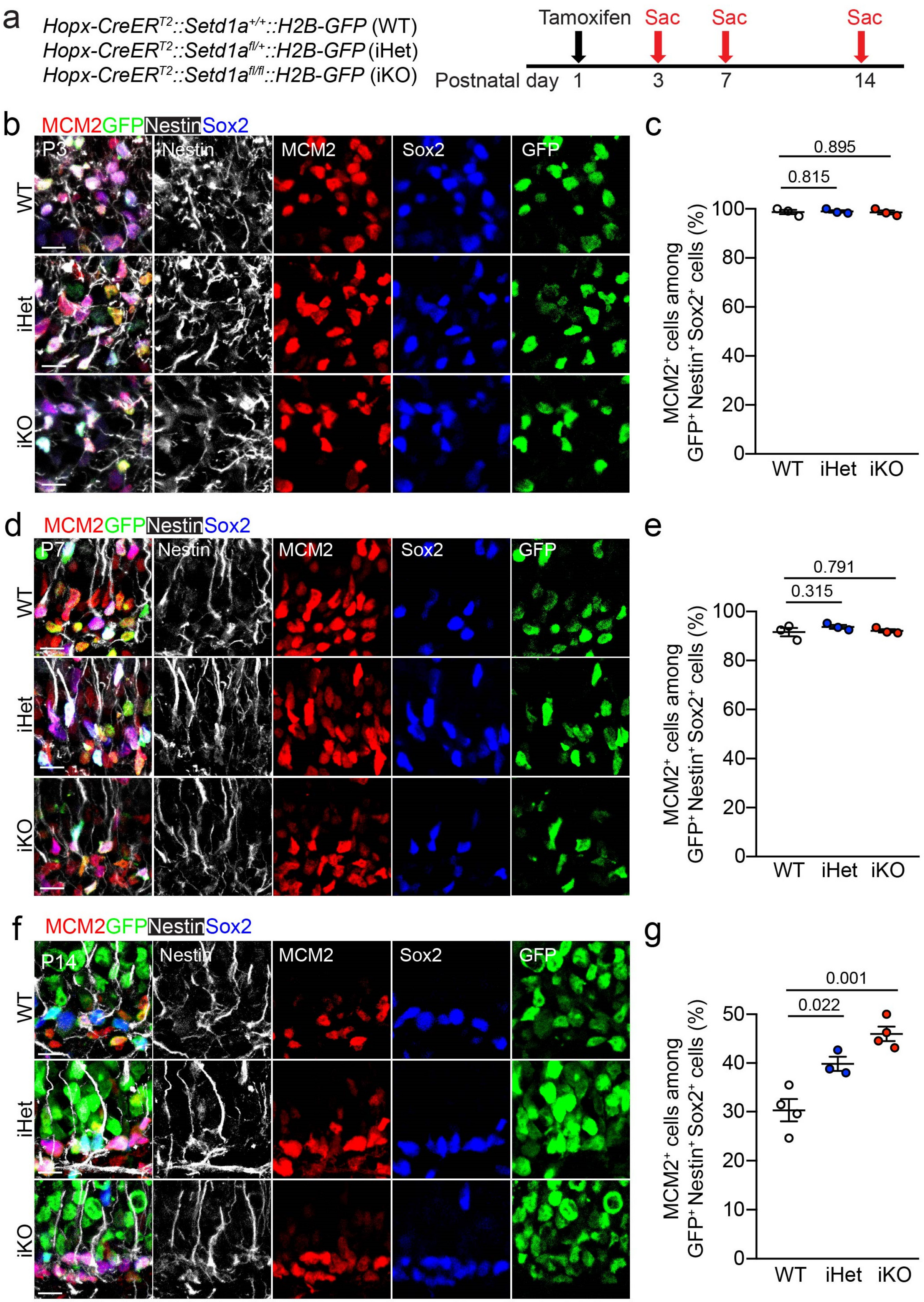

Supplementary Fig. 3. Deletion of *Setd1a* in NSCs in the developing DG. a A schematic diagram of the experimental design. **b-g** Sample confocal images of immunostaining for various

markers in WT, iHet and iKO DG at P3 (**b**), P7 (**d**) and P14 (**f**) and quantification of percentages of $MCM2^+Sox2^+Nestin^+GFP^+$ proliferating NSCs among $Sox2^+Nestin^+GFP^+$ total NSCs in the DG of WT, iHet and iKO mice at P3 (**c**), P7 (**e**) and P14 (**g**). Scale bars, 10 μm. Each dot represents data from one mouse. Values represent mean ± SEM (P3: n = 3/WT, 3/iHet, 3/iKO; P7: n= 3/WT, 3/iHet, 3/iKO; P14: n = 4/WT, 3/iHet, 4/iKO). Two-tailed Student's t test is used and the P values are shown. Source data are provided as a Source Data file.

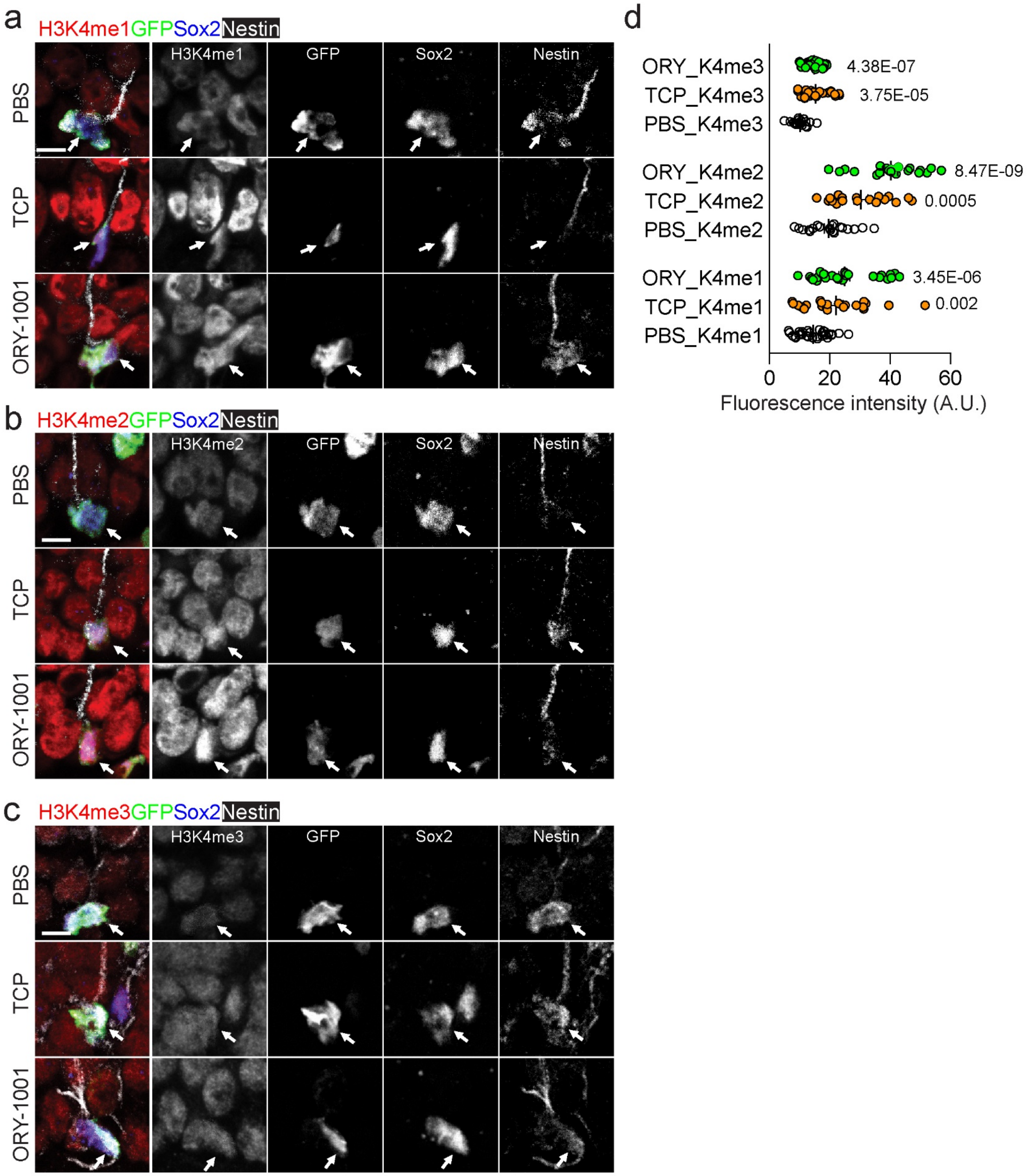

Supplementary Fig. 4. LSD1 inhibition increases H3K4 methylation levels in RGLs in the DG of adult WT mice. Shown are sample confocal images of immunostaining of GFP, Nestin, Sox2 and H3K4me1 (**a**), H3K4me2 (**b**) and H3K3me3 (**c**) (scale bars: 5 μm) and quantification of H3K4me1/2/3 levels in RGLs under the treatment of PBS (control), or LSD1 inhibitors (TCP or ORY-1001) in the DG of adult WT mice (**d**). Each dot represents data from one cell. Values represent mean ± SEM (H3K4me1: n = 30/PBS, 22/TCP, 32/ORY-1001; H3K4me2: n = 20/PBS, 18/TCP, 20/ORY-1001; H3K4me3: n = 18/PBS, 27/TCP, 30/ORY-1001; from n = 3 animals each

condition). A.U.: Arbitrary Unit. Two-tailed Student's t test is used and the P values are shown. Source data are provided as a Source Data file.

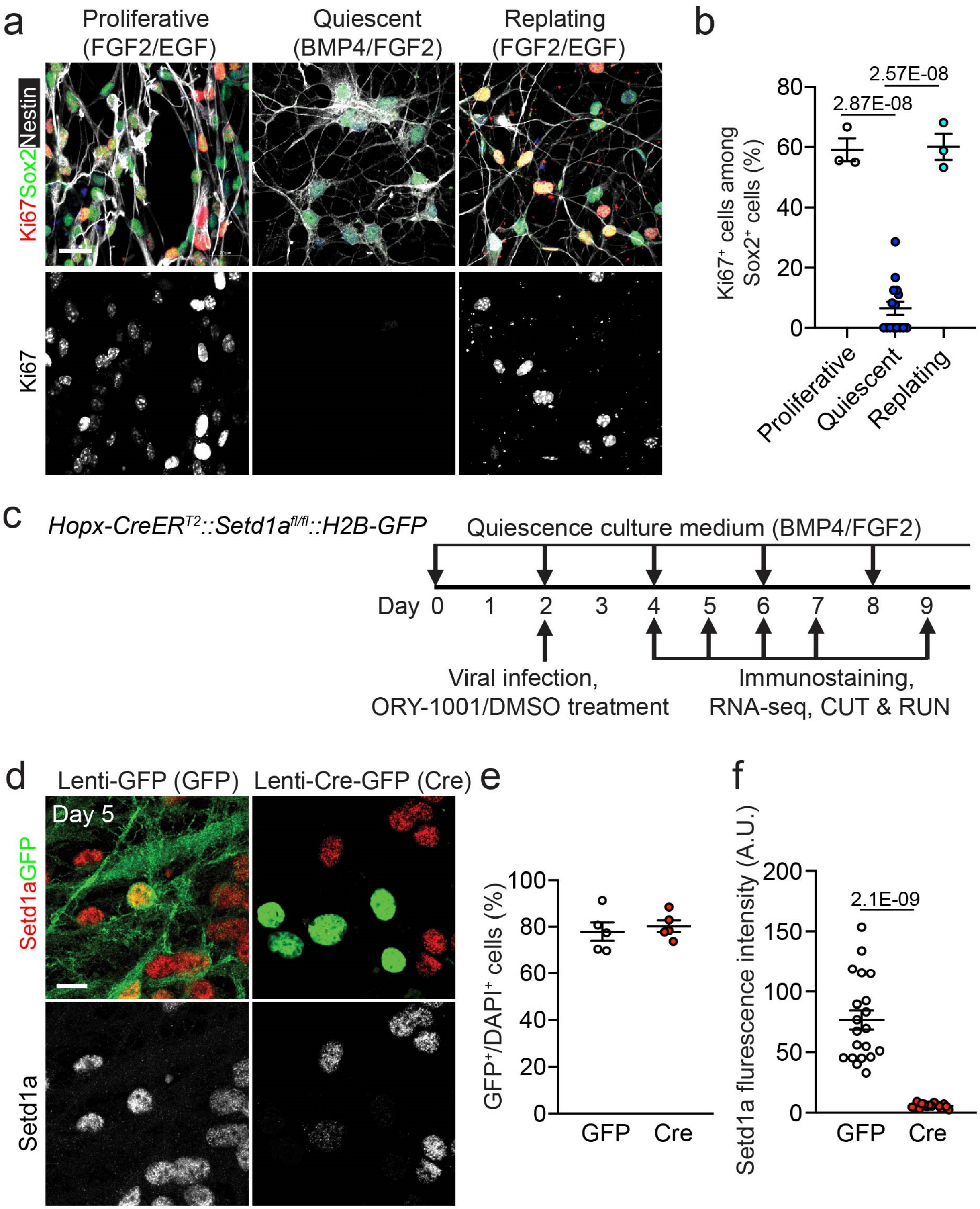

Supplementary Fig. 5. Analysis using quiescent adult NSCs in culture. a, b Adult NSCs derived from the DG of 7~8-week-old mice (*Hopx-CreER^{T2}::Setd1a$^{fl/fl}$::H2B-GFP*) were cultured in proliferative medium, quiescence medium, or after replating of cells from the quiescent medium into the proliferation medium. Shown are sample confocal images of immunostaining (**a**; scale bar: 20 μm) and quantification of percentages of Ki67$^+$Sox2$^+$ cells among Sox2$^+$ NSCs (**b**). Each dot represents data from one image. Values represent mean ± SEM (n = 3 independent cultures for each condition). **c** A schematic diagram of the experimental design. Cultured adult NSCs entered

the quiescent state after exposing cultures to the quiescence medium containing BMP4/FGF2. Quiescence medium was replaced every 2 days. At day 2, lentivirus expressing Cre-GFP or GFP alone was added to the culture medium. Next, cultures at day 4, 5, 6, 7, and 9 were harvested for RNA-seq, cultures at day 5, 7 and 9 were harvested for immunostaining and cultures at day 5 were harvested for Setd1a CUT & RUN. To inhibit LSD1 activity, ORY-1001, or DMSO as controls, was added to the quiescence medium at day 2, and the quiescence medium containing ORY-1001 was replaced every 2 days. **d-f** Effective deletion of *Setd1a* in cultured NSCs. Shown are sample confocal images of immunostaining at day 5 (**d**; scale bar: 10 μm) and quantification of percentages of GFP^+ infected cells over DAPI (**e**) and Setd1a levels in the infected control (GFP) and KO NSCs (Cre-GFP) (**f**). Each dot represents data from one culture (**e**) or one cell (**f**). Values represent mean ± SEM (e; n = 5 independent cultures for each condition). Two-tailed Student's t test is used and the P values are shown. A.U.: Arbitrary Unit. Source data are provided as a Source Data file.

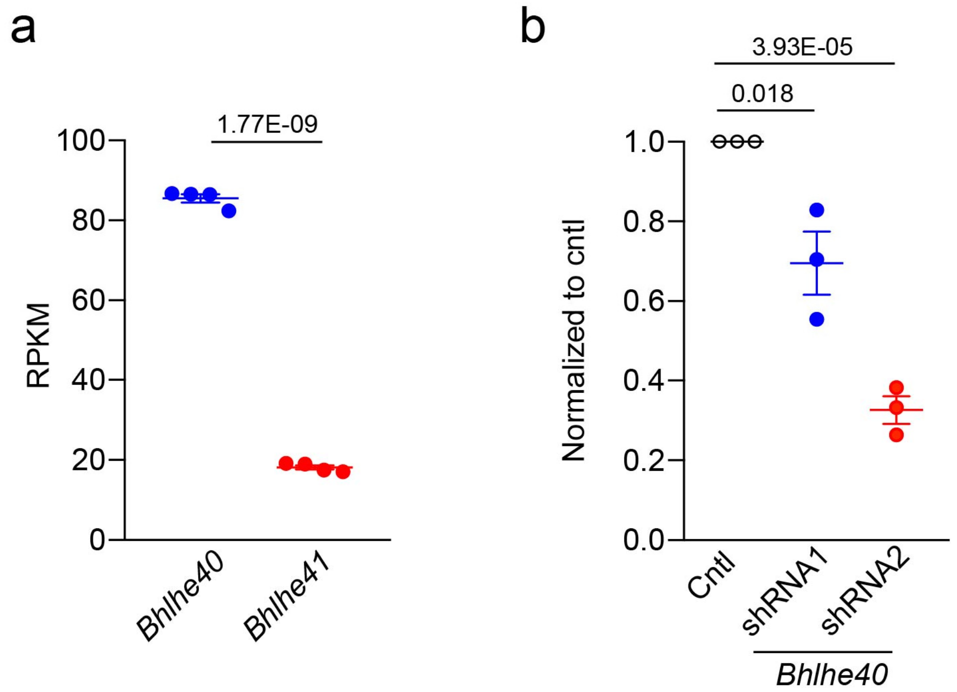

Supplementary Fig. 6. Expression of Bhlhe40 and Bhlhe41 in cultured quiescent adult NSCs and confirmation of efficacy of shRNAs against *Bhlhe40*. **a** RNA-seq analysis showing expression levels of *Bhlhe40* and *Bhlhe41* in cultured WT quiescent NSCs at day 5. Values represent mean ± SEM (n = 4 biological replicates for each condition). **b** qPCR analysis results showing effective knockdown of *Bhlhe40* by shRNA expression in cultured quiescent NSCs. Values were normalized to GAPDH and then control samples. Each dot represents data from one experiment. Values represent mean ± SEM (n = 3 for each condition). Two-tailed Student's t test is used and the P values are shown. Source data are provided as a Source Data file.

Whole gel images for Supplementary Fig. 1a

Setd1a

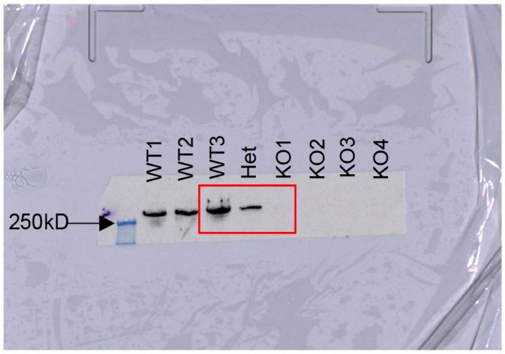

Tubulin

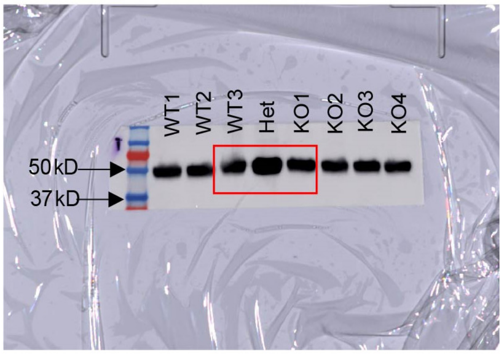

Supplement: Supplementary file 1 — Supplementary information [file 41467_2024_50010_MOESM1_ESM.pdf]
