# Supplementary material for: Epigenetic maintenance of adult neural stem cell quiescence in the mouse hippocampus via Setd1a
Source: Nat Commun. 2024 Jul 6;15:5674. doi: 10.1038/s41467-024-50010-y (PMC11227589; doi:10.1038/s41467-024-50010-y)
Supplement: Supplementary file 3 — Description of Additional Supplementary Files [file 41467_2024_50010_MOESM3_ESM.pdf]

SUPPLEMENTARY DATASETS (IN EXCEL FILES)

Supplementary Dataset 1. List of differentially expressed genes in cultured quiescent adult NSCs under different conditions.

Supplementary Dataset 2. List of locations of Setd1a CUT & RUN peaks in cultured WT quiescent adult NSCs and shared gene list between genes with a Setd1a peak at their promoters and genes with downregulated or upregulated expression in *Setd1a*-deficient adult NSCs.

Supplementary Dataset 3. List of antibodies and primers used in the current study.
