# Supplementary material for: Epigenetic maintenance of adult neural stem cell quiescence in the mouse hippocampus via Setd1a
Source: Nat Commun. 2024 Jul 6;15:5674. doi: 10.1038/s41467-024-50010-y (PMC11227589; doi:10.1038/s41467-024-50010-y)
Supplement: Supplementary file 7 — Reporting Summary [file 41467_2024_50010_MOESM7_ESM.pdf]

nature portfolio

Corresponding author(s): Guo-li Ming, Hongjun Song

Last updated by author(s): May 25, 2024

Reporting Summary

Nature Portfolio wishes to improve the reproducibility of the work that we publish. This form provides structure for consistency and transparency in reporting. For further information on Nature Portfolio policies, see our Editorial Policies and the Editorial Policy Checklist.

Statistics

For all statistical analyses, confirm that the following items are present in the figure legend, table legend, main text, or Methods section.

n/a	Confirmed	
☐	☒	The exact sample size (*n*) for each experimental group/condition, given as a discrete number and unit of measurement
☐	☒	A statement on whether measurements were taken from distinct samples or whether the same sample was measured repeatedly
☐	☒	The statistical test(s) used AND whether they are one- or two-sided<br>*Only common tests should be described solely by name; describe more complex techniques in the Methods section.*
☒	☐	A description of all covariates tested
☒	☐	A description of any assumptions or corrections, such as tests of normality and adjustment for multiple comparisons
☐	☒	A full description of the statistical parameters including central tendency (e.g. means) or other basic estimates (e.g. regression coefficient) AND variation (e.g. standard deviation) or associated estimates of uncertainty (e.g. confidence intervals)
☐	☒	For null hypothesis testing, the test statistic (e.g. *F*, *t*, *r*) with confidence intervals, effect sizes, degrees of freedom and *P* value noted<br>*Give P values as exact values whenever suitable.*
☒	☐	For Bayesian analysis, information on the choice of priors and Markov chain Monte Carlo settings
☒	☐	For hierarchical and complex designs, identification of the appropriate level for tests and full reporting of outcomes
☐	☒	Estimates of effect sizes (e.g. Cohen's *d*, Pearson's *r*), indicating how they were calculated

Our web collection on statistics for biologists contains articles on many of the points above.

Software and code

Policy information about availability of computer code

Data collection	Sequencing data was obtained from the NextSeq 550 sequencer (Illumina). Raw sequencing data were demultiplexed with bcl2fastq2 v2.17.1.14 (Illumina). Adaptors were trimmed using Trimmomatic v0.32 software. For RNA-seq, Trimmed sequence reads were mapped to mouse reference genome GRCm38 using STAR v2.5.2a. Only uniquely mapped reads were quantified at the gene level and summarized to gene counts using STAR-quantMode (GeneCounts), with multimapping and chimeric alignments discarded. For CUT&RUN, Trimmed sequence reads were uniquely mapped to mouse reference genome mm10 using Bowtie2 v2.1.0. Low mapping quality reads were filtered by SAMtools v0.1.18. Peak calling was performed using MACS2 v2.2.8.<br>StepOnePlus Real-Time PCR for qPCR acquisition; Amersham Imager 600 for western blot quantification
Data analysis	#### Bioinformatic analysis:<br>R (v3.6, RRID: SCR_001905); R Studio (v1.1, RRID: SCR_000432); ChIPseeker (RRID: SCR_021322); ComplexHeatmap (v2.18.0, RRID: SCR_017270); edgeR (v3.34.1, RRID: SCR_012802)<br><br>#### Image analysis:<br>Fiji Image J (NIH, v1.53f51, RRID:SCR_003070).<br><br>#### Making figures:<br>Adobe Illustrator CS6 (Adobe, v16.0.3, RRID: SCR_010279).

For manuscripts utilizing custom algorithms or software that are central to the research but not yet described in published literature, software must be made available to editors and reviewers. We strongly encourage code deposition in a community repository (e.g. GitHub). See the Nature Portfolio guidelines for submitting code & software for further information.

Data

Policy information about availability of data

All manuscripts must include a data availability statement. This statement should provide the following information, where applicable:

- Accession codes, unique identifiers, or web links for publicly available datasets
- A description of any restrictions on data availability
- For clinical datasets or third party data, please ensure that the statement adheres to our policy

The RNA-seq and CUT & RUN data generated in this study have been deposited in the NCBI's Gene Expression Omnibus database under accession code GSE250278 [https://www.ncbi.nlm.nih.gov/geo/query/acc.cgi?acc=GSE250278].Source data are provided with this paper. Any additional information required to reanalyze the data reported in this paper is available from the lead contact upon request.

Research involving human participants, their data, or biological material

Policy information about studies with human participants or human data. See also policy information about sex, gender (identity/presentation), and sexual orientation and race, ethnicity and racism.

Reporting on sex and gender	N/A
Reporting on race, ethnicity, or other socially relevant groupings	N/A
Population characteristics	N/A
Recruitment	N/A
Ethics oversight	N/A

Note that full information on the approval of the study protocol must also be provided in the manuscript.

Field-specific reporting

Please select the one below that is the best fit for your research. If you are not sure, read the appropriate sections before making your selection.

☒ Life sciences ☐ Behavioural & social sciences ☐ Ecological, evolutionary & environmental sciences

For a reference copy of the document with all sections, see nature.com/documents/nr-reporting-summary-flat.pdf

Life sciences study design

All studies must disclose on these points even when the disclosure is negative.

Sample size	Sample sizes were determined empirically based on prior experiments. For immunohistologial experiment at least three animals or three independent cell cultures was required for each group. For RNA-seq, four independent cultures for each group. For CUT&RUN, two independent cultures were used
Data exclusions	No data were excluded across experiments.
Replication	Data were reproducible across experiments.
Randomization	wild-type littermates were used as control for heterozygous and homozygous mice. Mice were randomly collected for experiments.
Blinding	Investigators were blinded in all immunohistological experiments during tissue processing, image acquisition, and cell quantification. Samples for RNAseq were blinded during the sequencing process.

Reporting for specific materials, systems and methods

We require information from authors about some types of materials, experimental systems and methods used in many studies. Here, indicate whether each material, system or method listed is relevant to your study. If you are not sure if a list item applies to your research, read the appropriate section before selecting a response.

Materials & experimental systems

n/a	Involved in the study
☐	☒ Antibodies
☐	☒ Eukaryotic cell lines
☒	☐ Palaeontology and archaeology
☐	☒ Animals and other organisms
☒	☐ Clinical data
☒	☐ Dual use research of concern
☒	☐ Plants

Methods

n/a	Involved in the study
☒	☐ ChIP-seq
☒	☐ Flow cytometry
☒	☐ MRI-based neuroimaging

Antibodies

Antibodies used

Chicken Polyclonal Anti-Nestin, Aves labs, Cat#NES, RRID: AB_2314882, IHC 1:500
Rabbit Polyclonal Anti-GFAP, Dako, Cat# Z0334, RRID: AB_10013382, IHC 1:500
Mouse Monoclonal Anti-GFAP, Sigma, Cat# MAB360, RRID: AB_11212597, IHC 1:500
Rabbit Monoclonal Anti-TBR2, Abcam, Cat#ab183991, RRID: AB_2721040, IHC 1:200
Guinea Pig Polyclonal Anti-DCX, EMD Millipore, Cat#AB2253, RRID: AB_1586992, IHC 1:200
Goat Polyclonal Anti-DCX, Santa Cruz, Cat# sc-8066, RRID: AB_2088494, IHC 1:200
Mouse Monoclonal Anti-Mcm2, BD Cat#610701, RRID: AB_398024, IHC 1:150
Rat Monoclonal Anti-Sox2, Invitrogen, Cat#14-9811-82, RRID: AB_11219471, IHC 1:150
Goat Polyclonal Anti-GFP, Rockland, Cat#600101215, RRID: AB_11181883, IHC 1:500
Goat Polyclonal Anti-mCherry, Biorbyt, Cat#orb11618, RRID: AB_2687829, IHC 1:500
Mouse Monoclonal Anti-Ki67, BD Biosciences, Cat#550609, RRID: AB_393778, IHC 1:150
Rabbit Polyclonal Anti-Setd1a, Abcam, Cat#ab70378, RRID: AB_1951955, Western Blot 1:150
Mouse Monoclonal Anti-Setd1a, Santa Cruz, Cat#sc-515590, RRID: N/A, IHC 1:150
Mouse Monoclonal Anti-α-Tubulin, Cell Signaling Technology, Cat#3873S, RRID: AB_1904178, Western Blot 1:5000
Rabbit Polyclonal Anti-H3K4me1, Abcam, Cat#ab8895, RRID: AB_306847, IHC 1:150
Rabbit Polyclonal Anti-H3K4me2, Abcam, Cat#ab7766, RRID: AB_2560996, IHC 1:150
Rabbit Polyclonal Anti-H3K4me3, Abcam, Cat#ab8580, RRID: AB_306649, IHC 1:150

Validation

All antibodies used in this study are commercial. Commercially available antibodies were validated by manufacturer for immunostaining and immunoblotting. Data are included on manufacturers web site and typically contain a western blot and example immunostaining. Citations are also included on the relevant web pages.

Eukaryotic cell lines

Policy information about cell lines and Sex and Gender in Research

Cell line source(s)

HEK293T cells were purchased from the ATCC.

Authentication

Authentication performed by ATCC and according to ATCC verification procedures, which includes Mycoplasma detection, STR profiling, and Sanger sequencing

Mycoplasma contamination

Cells were tested negative for mycoplasma.

Commonly misidentified lines
(See ICLAC register)

None

Animals and other research organisms

Policy information about studies involving animals; ARRIVE guidelines recommended for reporting animal research, and Sex and Gender in Research

Laboratory animals

Setd1afl/fl mice were generated in Dr. Suming Huang's lab (PMID: 25550471).
Nestin-Cre+/Tg mice were from Jackson Laboratory (003771) .
Hopx-CreERT2::H2B-GFP mice (PMID: 30929900) were generated by crossing Hopx-CreERT2 knock-in mice (Jackson Laboratory; 017606) that harbored a tamoxifen-inducible CreERT2 fusion gene with a Cre-reporter mouse line Rosa26flox-stop-flox-H2B-GFP mice (from lab of Dr. Z. Josh Huang) harboring loxP sites on either side of a STOP sequence and upstream of a fusion H2B-GFP protein cassette (PMID: 29684005).
Setd1afl/+ (WT) and Nestin-Cre::Setd1afl/+ (cHet); Strain: mouse, C57BL/6; Age: P14, P28, 3 months, 9 months
Hopx-CreERT2::Setd1a+/+:: H2B-GFP (WT), Hopx-CreERT2::Setd1afl/+::H2B-GFP (iHet) and Hopx-CreERT2::Setd1afl/fl::H2B-GFP (iKO); Strain: mouse, C57BL/6; Age: P1 and 6 weeks

Wild animals

No wild animals were used in this study.

Reporting on sex

Both male and female animals were used in the experiment. No obvious sex phenotype was observed in any of the experiments and data were combined

Field-collected samples	No field-collected samples were used in this study.
Ethics oversight	Animal procedures were performed in accordance with protocols approved by the Institutional Animal Care and Use Committee of the University of Pennsylvania.

Note that full information on the approval of the study protocol must also be provided in the manuscript.

Plants

Seed stocks	N/A
Novel plant genotypes	N/A
Authentication	N/A
