# Supplementary material for: Epigenetic maintenance of adult neural stem cell quiescence in the mouse hippocampus via Setd1a
Source: Nat Commun. 2024 Jul 6;15:5674. doi: 10.1038/s41467-024-50010-y (PMC11227589; doi:10.1038/s41467-024-50010-y)
Supplement: Supplementary file 2 — Peer Review File [file 41467_2024_50010_MOESM2_ESM.pdf]

nature portfolio

Peer Review File

Epigenetic maintenance of adult neural stem cell quiescence in the mouse hippocampus via Setd1a

Open Access This file is licensed under a Creative Commons Attribution 4.0 International License, which permits use, sharing, adaptation, distribution and reproduction in any medium or format, as long as you give appropriate credit to the original author(s) and the source, provide a link to the Creative Commons license, and indicate if changes were made. In the cases where the authors are anonymous, such as is the case for the reports of anonymous peer reviewers, author attribution should be to 'Anonymous Referee' followed by a clear attribution to the source work. The images or other third party material in this file are included in the article's Creative Commons license, unless indicated otherwise in a credit line to the material. If material is not included in the article's Creative Commons license and your intended use is not permitted by statutory regulation or exceeds the permitted use, you will need to obtain permission directly from the copyright holder. To view a copy of this license, visit http://creativecommons.org/licenses/by/4.0/.

REVIEWER COMMENTS

Reviewer #1 (Remarks to the Author):

Adult neurogenesis in hippocampal dentate gyrus (DG) requires neural stem cells (NSCs) to be maintained at a state of reversible cell cycle arrest called Quiescence. In this article, Zhao and Hong et al., have addressed a highly intriguing question: how is quiescence in adult neural stem cells (NSCs) maintained in adult DG? Using a set of rigorous experimental and analytical approaches and well-designed and executed functional experiments both in vitro and in vivo, authors have demonstrated that epigenetics mechanisms may play a role to maintain quiescence over the long term in the adult DG. Specifically, authors have identified that, Setd1a, a H3K4 methyltransferase is critical to sustain NSC quiescence in the adult DG. This study provides novel biological insights and will further our understanding of the molecular mechanisms underlying adult neurogenesis. I suggest the following minor modifications before publication.

1. Although the study revealed a Setd1a-dependent epigenetic mechanism that sustains NSC quiescence in the adult DG, authors should discuss whether other H3K4 methyl transferases (e.g., Setd1b, MLLs) could also play a role.

2. Authors have further shown that inhibition of LSD1, a histone demethylase, could alleviate Setd1a-deficient quiescent radial glial cells activation in the adult DG and increased levels of H3K4me1/2 and H3K4me3. Inhibition of LSD1 has also been reported to affect histone acetylation. The authors should discuss about specificity of LSD1 inhibitors and their limitations in the context of this study.

3. Authors have performed CUT and RUN assay to identify Setd1a-target genes and have identified 330 overlapped genes between the downregulated DEGs in Setd1a-deficit NSCs and genes harboring Setd1a peaks at promoters. The authors should also perform similar analysis on upregulated genes and report if there is any overlap between those DEGs and genes harboring Setd1a peaks at promoters.

Reviewer #2 (Remarks to the Author):

This is a mechanistic study of the role of the cell type specific role of the histone demethylase Setd1a. A key question that remains is how a broad epigenetic factor controls cell type specific cellular behavior, such as quiescence activation, through fine tune control of specific genes. Using both embryonic and adult specific deletion, the authors show: (1) Setd1a is required to maintain quiescence state of hippocampal neural stem cells at the developmental transition between proliferation and quiescence, (2) Setd1a continues to be required to maintain quiescence throughout adulthood, (3) the mechanism by which Setd1a maintains quiescence is through H3K4 methylation, (4) among the hundreds of Setd1a direct targets, one target, Bhlhe40, is also required for quiescence maintenance. The study is methodologically rigorous, including use of multiple conditional lines (Nestin-Cre and Hopx-CreERT2), use of a reporter gene to analyze cell intrinsic effects (Cre dependent H2B-GFP), use of both male and female mice, use of in vitro and in vivo models, validation of tools and controls (confirmation of Setd1a deletion via Western blot or IHC; confirmation of LSD1 inhibition; confirmation of Bhlhe40 knockdown). While this is not the first study to identify an epigenetic factor that regulates NSC quiescence, it does show a previously unidentified role of Setd1a in this process. There are some questions that need to be clarified to strengthen the impact of the work:

Major comments:

1. The authors mention the surprising discovery that adult mice with Setd1a haploinsufficiency developed an enlarged DG (Fig. 1). First, is Fig. 1b labeled correctly (the blue bar is cHet but this

means the GCL is reduced in cHet compared to WT), but the conclusion is that the DG is substantially enlarged in 3 month old cHet mice. Also, in Fig. 1b, what is the age of these mice (the figure legends says they are 3-month-old mice, but the blue bar is reduced).
2. Related to #1, in Supp. Fig. 1h, at 9 months, the blue bar is cHet and is larger than WT. Are these really the 9 mon or could it be switched with the 3 months. I would expect that by 9 months, the DG is not enlarged anymore, especially if the quiescent NSCs are depleted. Or does it take longer to see the reduction of DG after quiescent NSC depletion?
3. Related to #1 and #2, it is a bit confusing in the stated conclusion that there is an elevated activation of quiescent RGLs and increased neurogenesis, resulting in an increased number of DGCs and accelerated depletion of RGLs in the young adult DG which leads to decreased neurogenesis later in life. I think there are actually two separate conclusions here and this should be clearly indicated. For example, the first conclusion is deletion of Setd1a in all embryonic nervous system cells leads to an enlarged DG. The second conclusion is the RGLs in young adult mice are depleted over time.
4. The authors examine the % MCM2+ cells among Sox2+GFAP+Nestin+ cells in 3-month-old mice (Fig. 1h); to support the above conclusion of the elevated activation of quiescent RGLs and increased neurogenesis, followed by an accelerated depletion of RGLs in the young adult DG which leads to decreased neurogenesis later in life, the authors could also examine WT and cHet at P28 and 9 month old months since they have the tissues.
5. The RNA-seq and Cut & Run analysis suggest there is less expression of Bhlhe40 in Setd1a deficient quiescent NSCs and knockdown of Bhlhe40 in WT cells results in quiescence NSC activation, similar to iHet and iKO of Setd1a. The data also suggests Setd1a is enriched at promoters with downregulated DEGs, one of them being Bhlhe40. The authors could expand in the discussion a bit more about the epigenetic mechanism of Setd1a and H3K4 regulation of quiescence genes as well as how Bhlhe40 functions as a possible transcriptional regulator in this context. For example, why was Bhlhe40 singled out for downstream shRNA studies out of the 27? Were other genes tested or is there an effect of testing combinations of Setd1a targets. This could also be expanded in the discussion.

Reviewer #3 (Remarks to the Author):

In their manuscript “Epigenetic maintenance of adult neural stem cell quiescence in the mouse hippocampus via Setd1a2” Zhao and colleagues identified a novel function for Setd1a2 in regulating quiescence/activation of hippocampal progenitor cells. They show in vitro and in vivo how the histone methyltransferase Setd1a2 affects progenitor activation and convincingly define a novel epigenetic mechanism regulating stem cell quiescence. The experimental approach is straight-forward, the data are convincing and very complete (i.e., one may ask for in vivo cut & run experiments; but the data showing that an identified candidate indeed affects progenitor activation in vivo: clearly makes for that). Thus, we feel that this is an excellent study that merits publication. The only small change may be to add a short section in the discussion summarizing previously described functions of Setd1a2 in the context of stem cell activation/quiescence (e.g., in blood cells) that will be of interest to the reader.

We are glad that all three referees are quite positive about our work. **Reviewer 1** commented that our study "*addressed a highly intriguing question: how is quiescence in adult neural stem cells (NSCs) maintained in adult DG*" and stated that "*Using a set of rigorous experimental and analytical approaches and well-designed and executed functional experiments both in vitro and in vivo*"; "*This study provides novel biological insights and will further our understanding of the molecular mechanisms underlying adult neurogenesis*". **Reviewer 2** regarded our study as "*methodologically rigorous*"; and stated that our study "*show a previously unidentified role of Setd1a in this process*". **Reviewer 3** commented that our study "*convincingly define a novel epigenetic mechanism regulating stem cell quiescence*" and stated that "*The experimental approach is straight-forward, the data are convincing and very complete*" and "*this is an excellent study*".

We also appreciate the constructive comments from all three reviewers. We have followed their suggestions closely with new data and revisions of the text. We believe that our manuscript is improved.

Point-to-Point Responses

>>
Reviewer: 1

Adult neurogenesis in hippocampal dentate gyrus (DG) requires neural stem cells (NSCs) to be maintained at a state of reversible cell cycle arrest called Quiescence. In this article, Zhao and Hong et al., have addressed a highly intriguing question: how is quiescence in adult neural stem cells (NSCs) maintained in adult DG? Using a set of rigorous experimental and analytical approaches and well-designed and executed functional experiments both in vitro and in vivo, authors have demonstrated that epigenetics mechanisms may play a role to maintain quiescence over the long term in the adult DG. Specifically, authors have identified that, Setd1a, a H3K4 methyltransferase is critical to sustain NSC quiescence in the adult DG. This study provides novel biological insights and will further our understanding of the molecular mechanisms underlying adult neurogenesis. I suggest the following minor modifications before publication.

1. Although the study revealed a Setd1a-dependent epigenetic mechanism that sustains NSC quiescence in the adult DG, authors should discuss whether other H3K4 methyl transferases (e.g., Setd1b, MLLs) could also play a role.

We thank the reviewer for the suggestion. We discuss the potential role of other major H3K4 methyl transferases (Setd1b and MLLs) in regulating the quiescence of adult NSCs in the DG on Page 13.

2. Authors have further shown that inhibition of LSD1, a histone demethylase, could alleviate Setd1a-deficient quiescent radial glial cells activation in the adult DG and increased levels of H3K4me1/2 and H3K4me3. Inhibition of LSD1 has also been reported to affect histone acetylation. The authors should discuss about specificity of LSD1 inhibitors and their limitations in the context of this study.

We thank the reviewer for bringing up this concern. We discuss the specificity of LSD1 inhibitors, TCP and ORY-1001, on Page 7. We also discuss potential changes in histone acetylation caused by LSD1 inhibition on Page 13-14 in the revised manuscript.

3. Authors have performed CUT and RUN assay to identify Setd1a-target genes and have identified 330 overlapped genes between the downregulated DEGs in Setd1a-deficit NSCs and genes harboring Setd1a peaks at promoters. The authors should also perform similar analysis on upregulated genes and report if there is any overlap between those DEGs and genes harboring

Setd1a peaks at promoters.

We agree with the reviewer. We compared the genes harboring Setd1a peaks at promoters with the upregulated DEGs and identified 276 overlapped genes (revised Fig. 5c). We also provide the gene list of 276 overlapped genes in revised Supplementary Table 2.

>>
Reviewer: 2

This is a mechanistic study of the role of the cell type specific role of the histone demethylase Setd1a. A key question that remains is how a broad epigenetic factor controls cell type specific cellular behavior, such as quiescence activation, through fine tune control of specific genes. Using both embryonic and adult specific deletion, the authors show: (1) Setd1a is required to maintain quiescence state of hippocampal neural stem cells at the developmental transition between proliferation and quiescence, (2) Setd1a continues to be required to maintain quiescence throughout adulthood, (3) the mechanism by which Setd1a maintains quiescence is through H3K4 methylation, (4) among the hundreds of Setd1a direct targets, one target, Bhlhe40, is also required for quiescence maintenance. The study is methodologically rigorous, including use of multiple conditional lines (Nestin-Cre and Hopx-CreERT2), use of a reporter gene to analyze cell intrinsic effects (Cre dependent H2B-GFP), use of both male and female mice, use of in vitro and in vivo models, validation of tools and controls (confirmation of Setd1a deletion via Western blot or IHC; confirmation of LSD1 inhibition; confirmation of Bhlhe40 knockdown). While this is not the first study to identify an epigenetic factor that regulates NSC quiescence, it does show a previously unidentified role of Setd1a in this process. There are some questions that need to be clarified to strengthen the impact of the work:

Major comments:
1. The authors mention the surprising discovery that adult mice with Setd1a haploinsufficiency developed an enlarged DG (Fig. 1). First, is Fig. 1b labeled correctly (the blue bar is cHet but this means the GCL is reduced in cHet compared to WT), but the conclusion is that the DG is substantially enlarged in 3 month old cHet mice. Also, in Fig. 1b, what is the age of these mice (the figure legends says they are 3-month-old mice, but the blue bar is reduced).

We thank the reviewer for the careful reading and discovering our labeling mistake. We are sorry for the confusion. Fig. 1b shows data from 3-mon-old mice. The black line with a dot symbol represents WT, and the blue line with a square symbol represents cHet. We corrected this labeling error in the revised manuscript.

2. Related to #1, in Supp. Fig. 1h, at 9 months, the blue bar is cHet and is larger than WT. Are these really the 9 mon or could it be switched with the 3 months. I would expect that by 9 months, the DG is not enlarged anymore, especially if the quiescent NSCs are depleted. Or does it take longer to see the reduction of DG after quiescent NSC depletion?

We thank the reviewer for bringing up this concern. Supp. Fig. 1h shows data from 9-mon-old mice and it is correct. We found that DG was still enlarged in 9-month-old Setd1a cHet mice compared to their WT littermates. Our interpretation is that the enlargement is due to the generation of more granule neurons from quiescent RGL activation during the postnatal stages and such enlargement is maintained afterwards because of minimal cell death of granule neurons in adult mice. Indeed, we perform cleaved Caspase3 immunostaining and do not observe increased apoptosis in the cHet DG (data not shown). The number of quiescent RGLs in the adult DG is very small even compared to the number immature granule cells (see Fig. 1f) and their numbers contribute minimally to the

size of the DG. We did not examine the area of DG in mice older than 9 months since we did not maintain these animals longer than 9 months.

3. Related to #1 and #2, it is a bit confusing in the stated conclusion that there is an elevated activation of quiescent RGLs and increased neurogenesis, resulting in an increased number of DGCs and accelerated depletion of RGLs in the young adult DG which leads to decreased neurogenesis later in life. I think there are actually two separate conclusions here and this should be clearly indicated. For example, the first conclusion is deletion of Setd1a in all embryonic nervous system cells leads to an enlarged DG. The second conclusion is the RGLs in young adult mice are depleted over time.

We agree with the reviewer. We revised our conclusion on Page 5 following the suggestion.

4. The authors examine the % MCM2+ cells among Sox2+GFAP+Nestin+ cells in 3-month-old mice (Fig. 1h); to support the above conclusion of the elevated activation of quiescent RGLs and increased neurogenesis, followed by an accelerated depletion of RGLs in the young adult DG which leads to decreased neurogenesis later in life, the authors could also examine WT and cHet at P28 and 9 month old months since they have the tissues.

Thanks for the suggestion. We have performed the experiments and found that the percentage of active RGLs are higher in cHet than WT mice at both P28 and 9 months (revised Fig. 1h).

5. The RNA-seq and Cut & Run analysis suggest there is less expression of Bhlhe40 in Setd1a deficient quiescent NSCs and knockdown of Bhlhe40 in WT cells results in quiescence NSC activation, similar to iHet and iKO of Setd1a. The data also suggests Setd1a is enriched at promoters with downregulated DEGs, one of them being Bhlhe40. The authors could expand in the discussion a bit more about the epigenetic mechanism of Setd1a and H3K4 regulation of quiescence genes as well as how Bhlhe40 functions as a possible transcriptional regulator in this context. For example, why was Bhlhe40 singled out for downstream shRNA studies out of the 27? Were other genes tested or is there an effect of testing combinations of Setd1a targets. This could also be expanded in the discussion.

We thank the reviewer for the suggestions. We explained why we chose *Bhlhe40* for further study on Page 11. As described in the text, we focused on *Bhlhe40* for the following reasons: 1) our previous single-cell RNA-seq analysis revealed downregulation of *Bhlhe41* during RGL transition from quiescence to activation in the adult DG[1]. *Bhlhe40* is a paralogous gene of *Bhlhe41*, and Bhlhe40/41 form a homodimer or a heterodimer to function as transcriptional repressors[2]; 2) In our cultured quiescent adult NSCs, the expression level of *Bhlhe40* was much higher than that of *Bhlhe41* (Supplementary Fig. 6a); 3) Downregulation of *Bhlhe40* in *Setd1a*-deficient NSCs was rescued by LSD1 inhibition (Fig. 5f, g); 4) Depletion of *Bhlhe40* has been shown to activate quiescent muscle stem cells in the adult mice[3].

In the discussion section (Page 13), we mentioned the limitation of the current study in that we only validated the function of one target gene, *Bhlhe40,* from 27 potential targets. We agree with the reviewer that downregulation of *Bhlhe40* by Setd1a deletion is probably only one of many factors contributing to activation of quiescent adult NSCs. Therefore, we mentioned that "It is possible that *Setd1a* deficiency-induced activation of quiescent adult NSCs is a convergent effect of dysregulating multiple quiescence regulators."

In revised manuscript, we also expanded our discussion about potential mechanisms by which downregulation of *Bhlhe40* activates quiescent NSCs on Page 13. A previous study reported that loss of *Bhlhe40* elevated Notch signaling in muscle stem cells[3]. The Notch signaling pathway is essential for both the proliferative and quiescent states of NSCs. Notch promotes both proliferation and quiescence through dynamic expression of Notch effector proteins[4, 5, 6]. Probably,

downregulation of *Bhlh40* activates quiescent NSCs via disrupting Notch signaling.

>>>
Reviewer 3

In their manuscript "Epigenetic maintenance of adult neural stem cell quiescence in the mouse hippocampus via Setd1a2" Zhao and colleagues identified a novel function for Setd1a2 in regulating quiescence/activation of hippocampal progenitor cells. They show in vitro and in vivo how the histone methyltransferase Setd1a2 affects progenitor activation and convincingly define a novel epigenetic mechanism regulating stem cell quiescence. The experimental approach is straight-forward, the data are convincing and very complete (i.e., one may ask for in vivo cut & run experiments; but the data showing that an identified candidate indeed affects progenitor activation in vivo: clearly makes for that). Thus, we feel that this is an excellent study that merits publication. The only small change may be to add a short section in the discussion summarizing previously described functions of Setd1a2 in the context of stem cell activation/quiescence (e.g., in blood cells) that will be of interest to the reader.

We thank the reviewer for the suggestion. In revised manuscript, we discussed the functions of Setd1a in hematopoietic stem cells (HSCs)[7] on Page 13. Arndt and colleagues found that specific deletion of *Setd1a* in adult long-term (LT) HSCs resulted in G0-to-G1 transition and progression through cell cycle, and increased numbers of Ki67$^+$ LT-HSCs. These results suggest that *Setd1a* plays an important role in maintaining quiescence of adult somatic stem cell in different tissues.

References

1. Shin J*, et al.* Single-Cell RNA-Seq with Waterfall Reveals Molecular Cascades underlying Adult Neurogenesis. *Cell Stem Cell* **17**, 360-372 (2015).

2. Asanoma K*, et al.* Regulation of the Mechanism of TWIST1 Transcription by BHLHE40 and BHLHE41 in Cancer Cells. *Mol Cell Biol* **35**, 4096-4109 (2015).

3. Sun H*, et al.* Stra13 regulates satellite cell activation by antagonizing Notch signaling. *J Cell Biol* **177**, 647-657 (2007).

4. Imayoshi I, Sakamoto M, Yamaguchi M, Mori K, Kageyama R. Essential roles of Notch signaling in maintenance of neural stem cells in developing and adult brains. *J Neurosci* **30**, 3489-3498 (2010).

5. Sueda R, Imayoshi I, Harima Y, Kageyama R. High Hes1 expression and resultant Ascl1 suppression regulate quiescent vs. active neural stem cells in the adult mouse brain. *Genes Dev* **33**, 511-523 (2019).

6. Harris L, Guillemot F. HES1, two programs: promoting the quiescence and proliferation of adult neural stem cells. *Genes Dev* **33**, 479-481 (2019).

7. Arndt K*, et al.* SETD1A protects HSCs from activation-induced functional decline in vivo. *Blood* **131**, 1311-1324 (2018).

REVIEWERS' COMMENTS

Reviewer #1 (Remarks to the Author):

Zhao and Hong et al., have convincingly addressed previous concerns and suggestions. Overall, the manuscript is significantly improved, and the manuscript is a strong candidate for publication in Nature Communications.

Reviewer #2 (Remarks to the Author):

This is a beautiful study with mechanistic insight regarding the epigenetic regulation of adult neural stem cell quiescence. The additional data and discussion strengthen the ms's conclusions. I have no further concerns.

Reviewer #3 (Remarks to the Author):

The authors responded satisfyingly to our previous concerns/requests.

None
